# Dynamic regulation of the extracellular matrix in reward memory processes: a question of time

**DOI:** 10.3389/fncel.2023.1208974

**Published:** 2023-06-16

**Authors:** Jake Valeri, Barbara Gisabella, Harry Pantazopoulos

**Affiliations:** ^1^Department of Psychiatry and Human Behavior, University of Mississippi Medical Center, Jackson, MS, United States; ^2^Graduate Program in Neuroscience, University of Mississippi Medical Center, Jackson, MS, United States

**Keywords:** perineuronal net, extracellular matrix, substance use disorder, metabolic disorder, synaptic plasticity

## Abstract

Substance use disorders are a global health problem with increasing prevalence resulting in significant socioeconomic burden and increased mortality. Converging lines of evidence point to a critical role of brain extracellular matrix (ECM) molecules in the pathophysiology of substance use disorders. An increasing number of preclinical studies highlight the ECM as a promising target for development of novel cessation pharmacotherapies. The brain ECM is dynamically regulated during learning and memory processes, thus the time course of ECM alterations in substance use disorders is a critical factor that may impact interpretation of the current studies and development of pharmacological therapies. This review highlights the evidence for the involvement of ECM molecules in reward learning, including drug reward and natural reward such as food, as well as evidence regarding the pathophysiological state of the brain’s ECM in substance use disorders and metabolic disorders. We focus on the information regarding time-course and substance specific changes in ECM molecules and how this information can be leveraged for the development of therapeutic strategies.

## Introduction

Substance use disorders (SUD) are a debilitating group of psychiatric disorders that affect approximately 7% of people in the United States each year (SAMHSA, 2018; Grant et al., 2016). Relapse is a major factor limiting recovery from SUD and points to the strength of memory circuits involved in reward memory processing (Niaura et al., 1988; Carter and Tiffany, 1999; Weiss, 2005; Zironi et al., 2006; Janak and Chaudhri, 2010). Converging evidence from rodent models and human postmortem studies suggest a key role of extracellular matrix (ECM) molecules in the formation and maintenance of reward memories (Brown et al., 2007; Smith et al., 2011, 2015; Xue et al., 2014; Slaker et al., 2015, 2016a; Chioma et al., 2021; Seney et al., 2021; Browne et al., 2023). In addition, ECM abnormalities have been reported in several psychiatric disorders in which dysfunction in memory processing and synaptic regulation are key features, including schizophrenia (Eastwood and Harrison, 2006; Pantazopoulos et al., 2010, 2015; Enwright et al., 2016; Steullet et al., 2018), post-traumatic stress disorder (Gogolla et al., 2009), major depressive disorder (MDD) (Riga et al., 2017; Alaiyed et al., 2020; Blanco and Conant, 2021), and bipolar disorder (BD) (Fatemi et al., 2000; Pantazopoulos et al., 2015; Steullet et al., 2018) – each of which have significant comorbidity with SUD.

Involvement of the ECM in the regulation of reward memories may represent a shared feature across reward processes involved in strengthening memories necessary for survival such as food seeking. Several lines of evidence indicate that food and drugs of abuse activate overlapping brain circuitry (Blum et al., 2012; Volkow et al., 2013a). Furthermore, as the incidence of obesity and metabolic disorders continues to mount in the U.S., there has been an increasing interest in combining habitual overeating and drug abuse within a common diagnostic framework of disorders of addiction (Barry et al., 2009; Alsio et al., 2012; Blum et al., 2012; Baik, 2013; Volkow et al., 2013a,b). Growing evidence supports the involvement of ECM molecules in reward processes involved in high fat, high calorie diets that may contribute to metabolic disorders including obesity. The strength of reward memories contributes to relapse and habit forming, and targeting ECM processes to alleviate reward memory strength represents a promising strategy for the development of new treatments for SUD and obesity (Niaura et al., 1988; Carter and Tiffany, 1999; Lu et al., 2004; Weiss, 2005; Zironi et al., 2006; Janak and Chaudhri, 2010).

A growing number of studies demonstrate a dynamic regulation of the ECM during learning processes, including fear and reward learning (Gogolla et al., 2009; Slaker et al., 2016a). Evidence from over two decades of research indicates that the ECM is regulated by rewarding substances in a complex experience-dependent manner which may impact interpretation of the disease processes in SUD and metabolic disorders as well as treatment strategies. We summarize the current evidence for the involvement of the ECM in reward memory processes, SUD and obesity with an emphasis on how the ECM is modulated in a substance- and time-specific manner.

## The brain extracellular matrix

**FIGURE 1:**
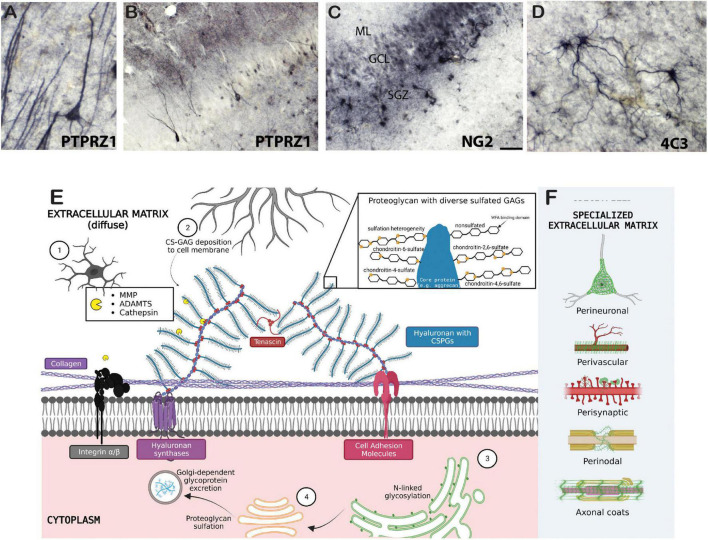
Biology of the extracellular matrix. Examples of chondroitin sulfate proteoglycan labeling in several cell types in the human brain include **(A,B)** immunohistochemical labeling of PTPRZ1 (phosphacan) neurons and glia in the human hippocampus; **(C)** NG2 labeled glial cells in the human hippocampus; and **(D)** 4C3 (specific CS sulfation motif) glial cell labeling in the human amygdala. **(E)** 1, microglia; 2, astrocyte; 3, rough endoplasmic reticulum (ER); 4, Golgi complex. Collagen proteins are tethered to the cell membrane by transmembrane integrins and other cell adhesion molecules. Cell adhesion molecules also attach to hyaluronan and CSPGs, which are attached to other CSPGs via tenascins. CSPGs are cleaved by endogenous proteases secreted by microglia, and are formed by intraneuronal rough-ER and Golgi-dependent processes. Further deposition of CS-glycosaminoglycans (GAGs) on neuron cell membranes is extraneously controlled astrocytes and oligodendrocytes. The zoomed in panel depicts sulfation heterogeneity of CS-GAGs as well as the non-sulfated binding domain of *Wisteria floribunda agglutinin* (WFA) lectin. **(F)** Specialized ECM structures are illustrated in green. Created with BioRender.com.

The brain extracellular matrix (ECM) is a network of chondroitin sulfate proteoglycans (CSPGs), heparan sulfate proteoglycans, glycoproteins, hyaluronan, and other molecules including axon guidance and cell adhesion molecules such as semaphorins and integrins (Table 1; Figure 1). In the brain, ECM molecules form several specialized structures including perineuronal nets (PNNs), perisynaptic ECM, perivascular ECM, and axonal coats (Schuster et al., 2001; Bruckner et al., 2008; Bekku et al., 2009; Dours-Zimmermann et al., 2009; Morawski et al., 2010; Baeten and Akassoglou, 2011; Lendvai et al., 2012; Susuki et al., 2013; Thomsen et al., 2017; Pantazopoulos et al., 2022; Tabet et al., 2022; Miguel-Hidalgo, 2023; Figure 1). These ECM structures are involved in a wide range of processes implicated in SUD including stabilization of synapses, axon guidance during neurodevelopment, regulation of diffusion of molecules such as neurotransmitters, ions, and metabolites, neuronal firing rates, receptor trafficking, protection from oxidative stress, and regulation of the blood-brain barrier (Hartig et al., 1999; Ishii and Maeda, 2008; Maeda, 2015). ECM molecules also have broad, complex roles in neurodevelopmental processes and brain injury [for reviews see Silver and Silver, 2014; Peters and Sherman, 2020; Carulli and Verhaagen, 2021; Fawcett and Kwok, 2022; Siddiqui et al., 2022; Schwartz and Domowicz, 2023]. CSPGs have been the primary focus of preclinical addiction studies and are one of the key proteoglycan families in the central nervous system (CNS). CSPGs are composed of a core protein with a varying number of covalently attached chondroitin sulfated glycosaminoglycan chains consisting of repeated pairs of glucuronic acid (GlcA) and N-acetyl-galactosamine (GalNAc) (Figure 1). The predominant CSPGs within the CNS are aggrecan, brevican, neurocan, phosphacan, neuron-glial antigen 2 (NG2), neuroglycan-C and versican (Milev et al., 1998; Deepa et al., 2006; Giamanco and Matthews, 2012). In addition to the specific functions of the core proteins, the glycosaminoglycan chains can vary in number and length, and are sulfated in various positions, which significantly contributes to the function of CSPGs (Asher et al., 2001; Wang et al., 2008; Maeda, 2010; Karus et al., 2012; Miyata et al., 2012; Alonge et al., 2019). For example, synaptic plasticity is regulated by chondroitin sulfation. CS-4 sulfation inhibits axonal growth (Smith-Thomas et al., 1995; Wang et al., 2008), whereas CS-6 is permissive for axonal growth (Lin et al., 2011; Miyata et al., 2012).

**TABLE 1:** Glossary.

Structural extracellular matrix molecules		
CSPG	Chondroitin sulfate proteoglycans	Aggrecan, brevican, neurocan, phosphacan, or versican core proteins with covalently attached glycosaminoglycan side chains
HSPG	Heparan sulfate proteoglycans	Agrin, syndecan, perlican, decorin, or glypican core proteins with covalently attached glycosaminoglycan side chains
	Hyaluronan	Large non-sulfated polysaccharides, linked to proteoglycans via hyaluronan and proteoglycan link proteins (HAPLN)
TN	Tenascins	Link hyaluronan-proteoglycan complexes to each other
PNN	Perineuronal nets	Composites of CSPG, hyaluronan, and tenascins condensed around neurons
GAG	Glycosaminoglycan	Long, negatively-charged, hetero sulfated chains of repeating disaccharide units
	Integrins	Signaling medium between the ECM and intracellular cytoskeleton
SEMA	Semaphorins	Axon guidance and cell morphology/Motility
NrCAM	Neural cell adhesion molecules	Fibronectin, laminin, focal adhesions, and integrins
**Endogenous proteases and extracellular matrix remodeling molecules**		
MMP	Matrix metalloproteinases	Large family of proteases which degrade a wide range of the ECM such as hyaluronan, CSPGs, HSPGs, collagen, etc.
ADAMTS	A disintegrin and metalloproteinase with thrombospondin motifs	Degrade specifically proteoglycans
	Cathepsins	Serine, cysteine, or aspartyl proteases which degrade specifically CSPGs
	Plasminogen	A precursor of plasmin, which is a broad range proteolytic enzyme capable of degrading a wide range of ECM proteins
tPA	Tissue plasminogen activator	Converters of plasminogen to its active proteolytic plasmin form
uPA	Urokinase plasminogen activator	
TIMP	Tissue inhibitors of metalloproteinases	Endogenous protease inhibitors that bind MMPs as their substrate
**Miscellaneous (non-endogenous)**		
	Doxycycline	Broad spectrum matrix metalloproteinase inhibitor
	FN-439	Broad spectrum matrix metalloproteinase and collagenase-1 inhibitor
ChABC	Chondroitinase ABC	Degrades glycosaminoglycan side chains
WFA	Wisteria floribunda agglutinin	Lectin which labels the terminal ends of chondroitin sulfate glycosaminoglycan residues, used in a majority of studies for quantification of perineuronal nets.

**FIGURE 2:**
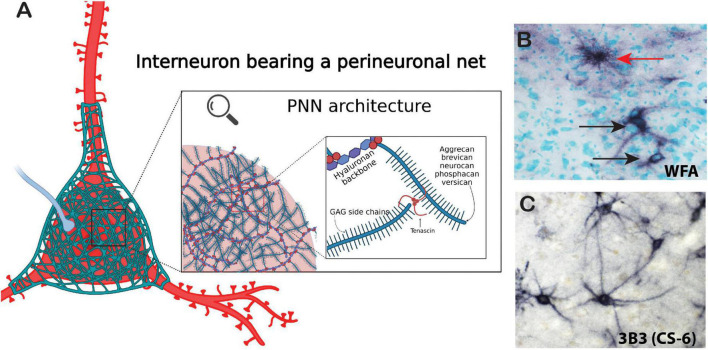
Architecture of the perineuronal net. Perineuronal nets are a meshwork of ECM components including chondroitin sulfate proteoglycan core proteins with varying numbers of CS-GAG chains, semaphorins, and tenascin-R. **(A)** Chondroitin sulfate core proteins are linked together by tenascins and to a hyaluronan backbone by hyaluronan link proteins (red spheres). **(B)** Immunohistochemical labeling of WFA (non-sulfated N-acetylgalactosamine). PNNs (black arrows) and astrocyte (red arrow) in the human brain. **(C)** Immunohistochemical labeling of the chondroitin-6-sulfation motif on PNNs in the human brain with labeled with the antibody 3B3. Created with BioRender.com.

Chondroitin sulfate proteoglycans form several specialized structures in the brain in addition to the diffuse ECM which exists around all cells within the CNS (Figure 1). PNNs represent the most well-studied of these structures. PNNs are composed of highly condensed ternary ECM molecules including CSPGs, hyaluronan, and tenascin and surround the soma, proximal dendrites, and axon initial segments of neurons, typically inhibitory fast-firing interneurons expressing parvalbumin (Figure 2; Hartig et al., 1994, 2022; Adams et al., 2001; Pantazopoulos et al., 2006). PNNs are involved in a broad range of functions including stabilization of synaptic plasticity, protection from oxidative stress, regulation of neuronal firing properties, N-methyl-D-aspartate (NMDA) receptor trafficking, and maintenance of ionic homeostasis (Kalb and Hockfield, 1988; Pizzorusso et al., 2002; Morawski et al., 2004; Sugiyama et al., 2008; Frischknecht et al., 2009; Gogolla et al., 2009; Dityatev et al., 2010; Gundelfinger et al., 2010; Frischknecht and Gundelfinger, 2012; Cabungcal et al., 2013; Wingert and Sorg, 2021). The role of PNNs as stabilizers of synaptic connections places them at the intersection of formation and consolidation of memories. PNNs are commonly labeled using the plant lectin *Wisteria floribunda agglutinin* (WFA), which binds to non-sulfated N-acetylgalactosamine residues at the terminal ends of CSPG saccharide chains (Hartig et al., 1994; Nadanaka et al., 2020). However, PNNs are complex structures with diverse composition, and labeling with antibodies directed against core CSPG proteins, glycoproteins, or specific sulfation motifs detects varying, partially overlapping populations of PNNs (Ajmo et al., 2008; Pantazopoulos et al., 2015; Dauth et al., 2016; Hartig et al., 2022; Scarlett et al., 2022). Several studies have demonstrated that the distribution of ECM molecules, including CSPGs and PNNs in the brain, varies greatly in a brain region and age specific manner (Mauney et al., 2013; Dauth et al., 2016; Ueno et al., 2017, 2018, 2019; Rogers et al., 2018; Mafi et al., 2020; Lupori et al., 2023).

Chondroitin sulfate proteoglycans also form several distinct structures in the brain in addition to PNNs (Figure 1). For example, ECM molecules including CSPGs condense as perisynaptic ECM aggregates on dendritic spines and contribute to synaptic homeostasis (Schuster et al., 2001). ECM molecules form perinodal structures at nodes of Ranvier along myelinated axons, where they contribute to axonal conductance through regulation of gap junctions by several processes including sodium channel clustering (Bekku et al., 2009; Dours-Zimmermann et al., 2009; Susuki et al., 2013; Pantazopoulos et al., 2022; Miguel-Hidalgo, 2023). CSPGs also interweave within myelin sheaths to form periaxonal aggregates called axonal coats (Bruckner et al., 2008; Morawski et al., 2010; Lendvai et al., 2012; Pantazopoulos et al., 2022). Furthermore, ECM molecules are critically involved in regulation of the blood-brain barrier and immune signaling through perivascular ECM structures (Baeten and Akassoglou, 2011; Thomsen et al., 2017; Tabet et al., 2022).

Recent studies demonstrate that the ECM, once thought to represent a stable component of the brain, is regularly modified in an activity-dependent manner through a complex combination of proteolytic remodeling and ECM recycling processes (Nagy et al., 2007; Brown et al., 2009; Ganguly et al., 2013; Dankovich and Rizzoli, 2022). Matrix metalloproteinases (MMP), a disintegrin and metalloproteinase with thrombospondin motifs (ADAMTS), and cathepsins are the putative endogenous proteases involved in degradation of ECMs (Page-McCaw et al., 2007; Mohamedi et al., 2020; Tran and Silver, 2021). These proteases are secreted by astrocytes, microglia, and neurons, indicating that individual neurons may, in part, be able to regulate the composition of their own PNNs (Rossier et al., 2015; Nguyen et al., 2020). MMPs, part of the metzincin superfamily, consist of several functional classes including collagenases, stromelysins, and gelatinases, with the gelatinases MMP-2, and MMP-9 and the stromelysin MMP-3 representing the predominant MMPs in the brain [for review see Rivera et al., 2010 and Brzdak et al., 2017]. ADAMTSs also serve as the substrate for tissue inhibitors of metalloproteinases (TIMP) which bind to these proteases and inhibit ECM proteolysis (Brew et al., 2000). Other proteolytic enzymes include tissue plasminogen activator (tPA) and urokinase plasminogen activator (uPA), which cleave and activate plasminogens that can degrade various ECM molecules, including proteoglycans (Saksela, 1985). Endogenous ECM proteases have been reported to modify the ECM during learning, which may facilitate formation of new synapses in response to environmental stimuli (Nagy et al., 2007; Brown et al., 2009; Ganguly et al., 2013). Recent studies have also demonstrated that ECM molecules, including PNNs, are modified in a diurnal manner, which may contribute to memory consolidation processes (Harkness et al., 2019; Pantazopoulos et al., 2020; Gisabella et al., 2021). Taken together, these studies suggest that the ECM is modified in a time-dependent manner which may impact interpretation of studies regarding the role of the ECM in SUD and the development of therapeutic strategies targeting the ECM. Within this context, we review the current literature regarding the involvement of the ECM in reward learning, including drugs of abuse and natural rewards such as high fat diet, and the current evidence for ECM pathology in SUD with an emphasis on time-course dependent findings.

## Dynamic extracellular matrix regulation by drugs of abuse

A number of studies have investigated the role of the ECM in animal models and human studies of SUD. Investigations have spanned an array of brain regions, including the prefrontal cortex (PFC) (Goldstein and Volkow, 2011), nucleus accumbens (NAc) (Quintero, 2013), ventral tegmental area (VTA) (Oliva and Wanat, 2016), hippocampus (Kutlu and Gould, 2016), amygdala (Warlow et al., 2017), cerebellum (Moulton et al., 2014), and hypothalamus (Zhang et al., 2020). Together, these brain regions are involved in regulating the rewarding and reinforcing effects of drugs, withdrawal symptoms, and the integration of memories of the rewarding effect of the drug and the associated context. Importantly, several studies cited throughout this review have varying definitions of the terms “acute” and “chronic.” For the purpose of this review, we define acute drug exposures as less than a period of 24-h (<2 h for “immediate”), and chronic drug exposure as greater than 3 consecutive daily sessions.

### Psychostimulants

Stimulant drugs such as cocaine and methamphetamine produce profound effects on CNS function including euphoria and increased motor activity/endurance (Nestler, 2005; Vazquez-Sanroman et al., 2015). Overuse of stimulant drugs can progress to the development of SUD and can also contribute to other serious conditions including psychosis, mood disorders, anxiety, and sleep/circadian rhythm dysfunction (American Psychiatric Association, 2013). Comorbidity of stimulant use with these disorders greatly complicates clinical interpretations and interventions for SUD as well as the comorbid conditions. Currently, there are no FDA-approved cessation pharmacotherapies for stimulant use disorder. Substantial evidence exists for a key role of the ECM in the formation and maintenance of stimulant use disorder endophenotypes (e.g., pervasive memories surrounding stimulant intoxication, withdrawal, and maintained use) (Table 2; Slaker et al., 2016a).

**TABLE 2:** Effects of psychostimulants on the ECM.

Treatment	Species	Brain area	Preharvest interval	ECM effect	Manipulation	Behavioral effect	References
**Acute psychostimulant exposure**							
Experimenter-administered cocaine	Mouse	NAc	30 min	↓β1-integrin	NA	NA	Wiggins et al., 2009
Cocaine IVSA	Mouse	NA	NA	NA	Heterozygous β3-integrin deficiency	↓ Cue-induced cocaine seeking	Garcia-Keller et al., 2019
				NA		↓ Enhancement of cue-induced cocaine seeking by MMP-9	
Cocaine CPP	Rat	NAc	NA	NA	Intra-NAc lentivirus upregulation of uPA	↑ CPP	Bahi et al., 2008
		NA	NA	NA	Inhibition of uPA with doxycycline	↓ Enhancement of CPP	
Nicotine conditioned place preference	Rat	HPC and PFC	3 h	↑ MMP-2 and MMP-9	NA	NA	Natarajan et al., 2013
Cocaine CPP and locomotor activity recordings	Mouse	NA	NA	NA	Heterozygous reelin deficiency	↑ Cocaine-induced hyperlocomotion	de Guglielmo et al., 2022
						No effect on CPP	
Experimenter-administered meth	Mouse	NA	NA	NA	Heterozygous reelin deficiency	No effect on meth-induced hyperlocomotion	Hume et al., 2020
Single cocaine injection	Rat	IL and PL PFC	2 h or 24 h	2 h: ↓ PNNs not apposing PVB neurons	NA	NA	Slaker et al., 2018
Single cocaine injection	Mouse	Amygdala, NAc, putamen	30 min	↑ tPA activity in amygdala	Homozygous tPA KO	Cocaine induces anxiolysis in tPA KO mice	Maiya et al., 2009
Single cocaine injection	Rat	HPC, NAc	1 day	↑*Sema3a* in hippocampus	NA	NA	Bahi and Dreyer, 2005
				↓*Sema3a* in NAc			
**Chronic psychostimulant exposure**							
Cocaine use disorder	Human	HPC	Postmortem	↓ MMP-9 protein	NA	NA	Mash et al., 2007
				↑β1-laminin and β6-integrin mRNA			
Experimenter-administered cocaine	Rat	IL and PL PFC	5 days: 2 h or 24 h	2 h: ↑ PNNs apposing PVB neurons 24 h: No effect	NA	NA	Slaker et al., 2018
Experimenter-administered cocaine (7 days)	Mouse	Deep cerebellar medial nucleus	24 h after last cocaine	↑ strong-type PNN labeling	NA	NA	Vazquez-Sanroman et al., 2015
Cocaine CPP	Rat	Cerebellum (lobule VIII of vermis)	8 cocaine pairings: 1.5 h after test (25.5 h after last cocaine)	NA	Intra-cerebellar chABC prior to CPP	No effect on CPP acquisition	Guarque-Chabrera et al., 2022
Cocaine CPP short-term memory (cue exposure)	Rat	Cerebellum, lobule VIII of vermis (LVIII)	6 days after last cocaine	NA	Intra-LVIII chABC prior to retest	↓ Cocaine short-term memory	Guarque-Chabrera et al., 2022
		Deep cerebellar nuclei (DCN)			Intra-DCN chABC prior to retest		
Stable nicotine IVSA	Rat	VTA and OFC	45 min after last session	*45 min:* ↓ PNNs apposing PVB neurons in VTA, OFC	NA	NA	Vazquez-Sanroman et al., 2017
			72 h after last session	*72 h:*↓ PNNs apposing PVB neurons in VTA, no effect in OFC			
Chronic (15 days) cocaine injections	Rat	HPC, NAc, VTA	24 h after last cocaine	↑ *Sema* mRNA in HPC, NAc, and VTA	NA	NA	Bahi and Dreyer, 2005
Cocaine RST from forced abstinence	Rat	HPC, VTA, NAc	24 h after last cocaine	↑ *Sema3a* in HPC	NA	NA	Bahi and Dreyer, 2005
				↓ *Sema3a* in NAc			
Chronic (13 days) cocaine and meth injections	Mouse	LH	Unknown	↑ HS disaccharide and sulfation content	NA	NA	Chen et al., 2017
Chronic (7 days) cocaine injections	Mouse	NAc	3 weeks since last cocaine	↑β1-integrin	NA	NA	Wiggins et al., 2009
Cocaine CPP	Rat	PL PFC	24 h	↑ PNNs apposing c-Fos neurons	NA	NA	Slaker et al., 2015
Chronic (5 days) meth injections	Rat	PFC, NAc, striatum, and VTA	2 h	↑ *Timp2* in PFC, NAc, and striatum	D1R and D2R antagonism	↓ Meth-induced *Timp* induction	Mizoguchi et al., 2007
Cocaine CPP RST	Rat	PFC and HPC	1, 3, 24 h	↑ MMP-9 and -2 in PFC at all timepoints, no changes in HPC	NA	NA	Brown et al., 2008
Cocaine CPP	Rat	PL PFC	24 h	↓ PNNs apposing c-Fos neurons in chABC animals	Intra-PL chABC before acquisition	↓ CPP	Slaker et al., 2015
Cocaine CPP RST	Rat	PL PFC	1 h	↓ PNNs apposing c-Fos neurons in chABC animals	Intra-PL chABC before memory reactivation	↓ CPP	Slaker et al., 2015
Cocaine EXT	Rat	PL PFC	∼14 days	No effect on PNNs apposing cFos neurons in chABC animals	Intra-PL chABC before extinction	No effect	Slaker et al., 2015
Cocaine IVSA RST	Rat	NAc	15 min after RST	↑ MMP-9 by cues for cocaine	NA	NA	Smith et al., 2014
Cocaine IVSA RST	Rat	NAc	15 min after RST	NA	FAK inhibitor	↓ Reinstatement of cocaine IVSA	Garcia-Keller et al., 2020
Meth cue exposure during abstinence	Rat	NAc	30 min after cued relapse	Increased MMP-2,9 by meth cues	NA	NA	Lewandowski et al., 2023
Cocaine CPP and IVSA	Rat	LH	NA	NA	Intra-LH chABC injection before behavior	↓ CPP and IVSA	Blacktop et al., 2017

Acute (top) and chronic (bottom) effects of cocaine, methamphetamine, and nicotine on ECM molecules. Preharvest interval denotes the time between the most recent drug exposure and euthanasia. CPP, conditioned place preference; IVSA, intravenous drug self-administration; RST, reinstatement; NAc, nucleus accumbens; HPC, hippocampus; PFC, prefrontal cortex; IL, infralimbic; PL, prelimbic; VTA, ventral tegmental area; LH, lateral hypothalamus; OFC, orbitofrontal cortex; MMP, matrix metalloproteinase; chABC, chondroitinase ABC; PNN, perineuronal net; PVB, parvalbumin; SEMA, semaphorin; TIMP, tissue inhibitor of metalloproteinase; tPA, tissue plasminogen activator; HS, heparan sulfate; D1R/D2R, dopamine 1 and 2 receptors, FAK; focal adhesion kinase.

#### Acute stimulant exposure

An intriguing collection of studies have examined the acute response of ECM molecules to psychostimulant drugs. Integrins serve as a tether for cell membrane-bound condensed ECM and also are involved in cytoskeleton signaling cascades that create a bidirectional interplay of the local ECM and intraneuronal signaling pathways, sometimes serving as the substrate for MMPs (ffrench-Constant and Colognato, 2004; Mezu-Ndubuisi and Maheshwari, 2021; Samuel et al., 2023). Temporally, β1-integrin protein levels in the NAc of mice are significantly decreased 30 min after a cocaine injection, but not at zero or 120 min (Wiggins et al., 2009). Conditional knockdown of β3 integrins in the mouse NAc during cocaine self-administration training prevents cue-induced cocaine seeking and transient excitatory synaptic potentiation (Garcia-Keller et al., 2019), suggesting that the acute increase of β-integrins may be involved in formation of cocaine associated memories. Activating endogenous ECM proteases, such as MMPs, using tPA increases cue-induced cocaine seeking (Garcia-Keller et al., 2019). In line with this, lentivirus-mediated upregulation of uPA (another endogenous MMP activator) in rats potentiates cocaine place preference acquisition (Bahi et al., 2008). Interestingly, knockdown of β3-integrin ameliorates the effect of tPA-induced seeking (Garcia-Keller et al., 2019). Decreased integrin during reward learning may not only inhibit the consolidation of cocaine-associated cues, but also the functions of neurons that are typically ensheathed by PNNs. Another study in rats measured hippocampal MMP protein levels 3 h following nicotine place preference conditioning, demonstrating relatively mild increases of MMP-2 and -9 during the early training sessions, and a much greater nicotine-induced MMP-9 upregulation during later conditioning sessions (Natarajan et al., 2013). Importantly, nicotine acts through mechanisms which are distinct from other psychostimulants of abuse (i.e., cholinergic stimulation of mesolimbic dopamine) and its effects as a reinforcer rely heavily on learning the cues paired with its administration (McClernon et al., 2016). Thus, progressively increased MMP induction over the course of nicotine CPP training may reflect contextual learning mechanisms induced by nicotine.

While acute exposure to stimulants appears to upregulate many ECM proteases, PNN fluorescent intensity 2 h after initial cocaine exposure is significantly decreased in the rat PFC coinciding with increased inhibitory glutamic acid decarboxylase- (GAD) 65/67 synaptic puncta on PNN-coated parvalbumin neurons (Slaker et al., 2018). Reduced PNN composition at 2 h corresponds with findings from a prior study which identified increased expression of tPA, an upstream promoter of MMPs (Tsuji et al., 2005), 30 min following a cocaine injection (Maiya et al., 2009). In comparison, differential effects of semaphorin 3a, a related ECM molecule, were observed between the rat hippocampus and NAc (Bahi and Dreyer, 2005). One day after receiving a bolus cocaine injection, expression of semaphorin 3a, a chemorepulsive protein involved in regulating PNN plasticity (Dick et al., 2013; de Winter et al., 2016), is significantly increased in the hippocampus, but decreased in the NAc (Bahi and Dreyer, 2005). Semaphorin 3a binds to the 4,6-sulfated CS in PNNs and decreased semaphorin 3a is associated with enhanced plasticity of neurons surrounded by PNNs (Dick et al., 2013; de Winter et al., 2016), suggesting that PNNs in these regions are differentially regulated by acute cocaine exposure. Further studies regarding brain region-specific changes in PNN components as well as perisynaptic ECM and axonal coats following acute exposure to psychostimulants will provide insight into the specific role of the ECM in synaptic plasticity during psychostimulant use.

#### Chronic stimulant use

Extracellular matrix molecules have been extensively studied during chronic stimulant models of self-administration, extinction, and reinstatement. Additionally, effects of PNN digestion using the exogenous enzyme chondroitinase ABC (chABC) have been examined at various timepoints across drug administration protocols by several groups [for review see Slaker et al. (2016a)]. Neuroinflammation is a hallmark of chronic stimulant use, and is induced by direct actions of these drugs on microglia resulting in upregulated inflammatory cytokines (Kohno et al., 2019). However, evidence suggests that stimulant use does not result in persistent microgliosis (Narendran et al., 2014). There is a current lack of evidence regarding how neuroinflammation and ECM alterations intersect in the acute vs. chronic phases of stimulant use disorder.

Protracted exposure to cocaine increases the numerical density and intensity of PNNs in several brain regions, such as the PFC (prelimbic/infralimbic cortex) and cerebellum, whereas nicotine decreases numerical densities of PNNs in the VTA and orbitofrontal cortex (Slaker et al., 2015, 2018; Vazquez-Sanroman et al., 2015, 2017; Guarque-Chabrera et al., 2022). Increased expression of several semaphorin genes was reported in the hippocampus, NAc, caudate putamen, and VTA of rats during chronic cocaine exposure and relapse (Bahi and Dreyer, 2005), suggesting that semaphorin expression may contribute to increased PNNs in these regions during chronic cocaine exposure. In addition to changes in PNNs and semaphorins, HSPGs, such as syndecan-3, are altered after prolonged exposure to cocaine and methamphetamine, increasing both in disaccharide content and total sulfation (Chen et al., 2013, 2017). Interestingly, another study reported no significant effect of 5 days of cocaine exposure on sulfation of diffuse CS, HS or hyaluronan (Slaker et al., 2018). The composition and functional properties of HS and CS proteoglycan/GAG families have a great deal of similarity, both subserving synaptic homeostasis and acting as structural chemorepellent barriers to neuroplasticity (Maeda et al., 2011). β1-integrin protein levels also undergo a ∼1.5-fold increase after prolonged withdrawal from chronic cocaine exposure (Wiggins et al., 2009). Further, expression of tissue inhibitors of MMPs (TIMPs) is significantly upregulated following repeated methamphetamine exposures (Mizoguchi et al., 2007) indicating that suppression of MMPs after prolonged stimulant use may reduce PNN degradation and contribute to the reported increases in PNNs following chronic drug use. In comparison, multiple studies converge on upregulation of proteolytic activity, including increased MMP expression, upon re-exposure to either stimulant-associated cues (i.e., stimulus lights in self-administration chambers, drug-paired CPP chambers) or stimulants alone (Brown et al., 2007, 2008; Smith et al., 2014; Garcia-Keller et al., 2020; Lewandowski et al., 2023). Importantly, the increase in inhibitory (GAD-65/67) synaptic puncta in the PFC during acute cocaine exposure is retained throughout chronic exposure (Slaker et al., 2018). Removal of PNNs using chABC in the anterior dorsal lateral hypothalamus, amygdala or PFC inhibits stimulant-induced place preference and reinstatement of stimulant self-administration (Xue et al., 2014; Slaker et al., 2015; Blacktop et al., 2017; Marchant, 2019). A human postmortem study of the hippocampus in subjects with history of cocaine use identified significantly decreased protein levels of the active form of MMP-9, along with a corresponding upregulation of several cell adhesion genes (Mash et al., 2007). Taken together, studies on stimulants suggest a biphasic modification of ECM over the course of stimulant use. MMPs increase during the acute phase of drug exposure, presumably to degrade PNNs to allow the formation of new synaptic connections involved in reward memory encoding. After prolonged use, MMPs decrease in activity which contributes to decreased modification of CSPGs and HSPGs and in turn the reported increase in PNN composition, which may stabilize the newly formed synapses involved in reward memory and contribute to relapse.

### Opioids

The significant abuse liability and mortality of opioids has gained attention over the past couple of decades due to the rise in overdose deaths from illicit and synthetic opioid analogs, particularly heroin and fentanyl (Spencer et al., 2022). Prescription opioids are highly effective pain-relieving medicines; however, they can also lead to dependence, resulting in misuse and development of opioid use disorder (OUD). Thus, the development of new prescription opioids, or additives to opioids, that retain analgesic qualities and reduce the risk of dependence is a current research focus (Negus and Freeman, 2018). A majority of heroin users report using opioids for the first time early in life (Jones, 2013), a critical period of brain circuit wiring where ECM molecules are involved in shaping later stage maturation of brain circuits including the formation of PNNs (Pizzorusso et al., 2002; Gogolla et al., 2009; Mauney et al., 2013; O’Connor et al., 2019; Nguyen et al., 2020; Gisabella et al., 2021). Furthermore, several lines of evidence propose a critical role of the ECM in the analgesic and rewarding effects of opioids (Table 3; Kruyer et al., 2020; Ray et al., 2022).

**TABLE 3:** Effects of opioids on the ECM.

Treatment	Species	Brain area	Preharvest interval	ECM effect	Manipulation	Behavioral effect	References
**Acute opioid exposure**							
Morphine and EM-2 incubation *in vitro*	MCF-7 breast cancer cell line	NA	3–72 h in culture	Concentration-gradient ↓ of *Mmp2* and *Mmp9* by morphine and EM-2	NA	NA	Gach et al., 2011
Heroin IVSA cued RST	Rat	NAc	0 min	Increased MMP-9 puncta around D1R MSNs	MMP-2 and MMP-9 inhibitors	MMP-9 inhibitors decrease cued reinstatement	Chioma et al., 2021
Morphine incubation *in vitro*	Human brain microvascular endothelial cells	NA	24 h	↓ MMP-2, LAMA-4	NA	NA	Vujic et al., 2022
**Chronic opioid exposure**							
Heroin IVSA, EXT and RST	Rat	mPFC, NAc, striatum	21 days (EXT)	EXT: ↓ TNR and BCAN in NAc, ↓ 145 kDa BCAN in mPFC	i.c.v. FN439 injection	Decreased cue-induced heroin RST	Van den Oever et al., 2010
			0 min (RST)	RST: ↓ TNR in NAc			
Heroin IVSA RST	Rat	NAc	15 min after RST	↑ MMP-9 by cue exposure	NA	NA	Smith et al., 2014
Escalating morphine and naloxone mediated withdrawal	Rat	Spinal cord (laminae I-VII)	48 h	Significant increase of MMP-9 in morphine treated animals	Intrathecal MMP-9 inhibition	↓ Morphine withdrawal	Liu et al., 2010
						No effect on pain-threshold or morphine-induced analgesia	
Heroin IVSA forced abstinence	Rat	IL-PFC and vOFC	1 day	1 day: ↑ PNNs in IL and OFC	NA	NA	Roura-Martinez et al., 2020
			30 d	30 days: No PNN changes compared to controls			
Opioid use disorder	Human	DL-PFC and NAc	Postmortem	↑ Differentially expressed transcripts related to CS-GAG biosynthesis in DL-PFC, NAc	NA	NA	Seney et al., 2021
Opioid use disorder	Human	Midbrain	Postmortem (overdose)	↑ IL-4 in opioid overdoses	NA	NA	Wei et al., 2023

Acute (top) and chronic (bottom) effects of opioids on ECM molecules. Preharvest interval denotes the time between the most recent drug exposure and euthanasia. CPP, conditioned place preference; IVSA, intravenous drug self-administration; NAc, nucleus accumbens; HPC, hippocampus; PFC, prefrontal cortex; IL, infralimbic; PL, prelimbic; VTA, ventral tegmental area; LH, lateral hypothalamus; OFC, orbitofrontal cortex; MMP, matrix metalloproteinase; chABC, chondroitinase ABC; PNN, perineuronal net; TNR, tenascin-R; LAMA, laminin; tPA, tissue plasminogen activator; HS, heparan sulfate; D1R/D2R, dopamine 1 and 2 receptors; MSN, medium spiny neuron; IL, interleukin.

#### Acute opioid exposure

There is extensive evidence suggesting that the ECM in the CNS is altered by pain, Nakamoto et al. (2012); Ishiguro et al. (2014); Tajerian and Clark (2015); Hu et al. (2021); Mascio et al. (2022); Tansley et al. (2022). Together these studies indicate that microglia degrade PNNs in the spinal cord when pain levels are high to disinhibit the spinal cord nociceptive afferents that evoke pain perception. Chronic pain results in an increased abundance of PNNs, possibly to stabilize newly formed synapses involved in the discriminative and emotional aspects of pain (Mascio et al., 2022). There is, however, relatively limited evidence regarding the immediate effects (0 to 2 h) of opioids on the ECM, particularly regarding the potential role of the ECM as it pertains to pain relief processes by opioids in multiple modalities of nociception (e.g., thermal, inflammatory, neuropathic). Studies indicate that acute administration of morphine dose-dependently decreases MMP-2, MMP-9, and laminin-4 (LAMA-4) expression in both breast cancer and human brain microvascular endothelial cell lines from 3 h up to 72 h (Gach et al., 2011; Vujic et al., 2022). Speculatively, the 3-h timepoint examined in cell lines may capture a period after prior increases in MMP expression, considering that levels of laminins are also decreased. In line with this, MMP-2 and -9 display increased activity around dendritic spines of dopamine 1 (D1) receptor- medium spiny neurons in the rat NAc during heroin reinstatement (Chioma et al., 2021). Rapid proteolytic induction may also reflect opioid activation of interleukin-33 expression and intracellular microglial cascades downstream of the IL1RL1/ST2 receptor involved in memory processing, which would increase expression of several ECM proteases to facilitate synaptic plasticity (Nguyen et al., 2020; Hu et al., 2021). Alternatively, acute decreases of MMP activity in response to opioids may contribute to their mechanism of analgesia, as acute pain is associated with increased MMP expression and degradation of the ECM (Tansley et al., 2022). Whether effects on the ECM incurred by opioids in the spinal cord and brain are congruent or divergent is yet to be determined. It is possible that opioids have regional effects on the ECM. For example, in the brain, opioids may contribute to synaptic plasticity underlying drug-associated memories, whereas in the spinal cord, opioids may act on the ECM to inhibit excitatory nociceptive afferents. As evidence in this field continues to mount, it will be important for future studies to improve our understanding of the temporal ECM changes in response to opioids and how they interact with the effect of pain on the ECM.

#### Chronic opioid use

Early work examined ECM molecules in the brain of rats following heroin self-administration (Van den Oever et al., 2010). Acute abstinence from opioids (1 day) is associated with an increased numerical density of PNNs in the PFC. Long-term abstinence, however, (21-30 days), is associated with a decrease in protein levels of the PNN components tenascin-R and brevican in the medial PFC and NAc, but no significant difference in WFA labeled PNN numerical densities compared to control animals (Van den Oever et al., 2010; Roura-Martinez et al., 2020). This suggests that PNN composition may be modified, or the changes in tenascin-R and brevican occur on other ECM structures distinct from PNNs. Alternatively, multiple studies point to upregulation of MMPs in the NAc during cue-induced heroin relapse (Liu et al., 2010; Smith et al., 2014; Chioma et al., 2021). Interestingly, inhibiting PNN degradation during reinstatement with intracerebroventricular FN-439 administration or enhancing PNN degradation during extinction with chABC in the amygdala impairs cue-induced heroin reinstatement (Van den Oever et al., 2010; Xue et al., 2014).

Two recent human postmortem studies provide critical evidence regarding alterations of ECM and neuroimmune gene expression pathways in the brain of people with OUD (Seney et al., 2021; Wei et al., 2023). Transcriptional profiling of the dorsolateral PFC and NAc revealed a shared, significant upregulation of transcripts related to CSPG and GAG biosynthesis in these two brain regions in subjects with OUD (Seney et al., 2021). Notably, pathways involved in cytokine-mediated inflammation and synapse remodeling were also upregulated (Seney et al., 2021). Moreover, a recent postmortem study of the ventral midbrain transcriptome identified significant upregulation of the IL-4 receptor in microglia in individuals who died by opioid overdose, providing further support for neuroinflammation in the brain of people with OUD (Wei et al., 2023). Collectively, studies on the effects of opioids on brain ECM molecules highlight an interplay of the brain’s immune system and the ECM in regulating both reward stabilization and analgesia.

### Alcohol

Alcohol use disorder (AUD) is the most prevalent form of SUD in the U.S. (American Psychiatric Association, 2013). Chronic, heavy alcohol use results in an array of health issues affecting several organ systems including the brain and increases mortality risk. A growing number of studies have investigated ECM alterations in response to alcohol early in adolescence, as well as acute, and chronic alcohol use (Table 4; Lasek, 2016).

**TABLE 4:** Effects of alcohol on the ECM.

Treatment	Species	Brain area	Preharvest interval	ECM effect	Manipulation	Behavioral effect	References
**Acute alcohol exposure**							
Daily exposure	Rat	HPC, PFC cerebellum	2, 4, and 6 days	Progressive MMP-9 ↓	Morris water maze	↓ Performance in animals with low *Mmp9*	Wright et al., 2003
EtOH vapor induced-alcohol SA escalation	Rat	NA	NA	NA	i.c.v. FN-439 during withdrawal	No escalation of self-administration	Smith et al., 2011
CPP	Mouse	HPC	NA	NA	Adenovirus MMP-9 upregulation before CPP training	↓ Place preference	Yin et al., 2023
DID (1 wk)	Mouse	Insula	20 h	No effect on PNNs, ACAN BCAN, or PCAN	NA	NA	Chen et al., 2015
**Chronic alcohol exposure**							
Adolescent intermittent alcohol exposure	Mouse	OFC	73 d	↑ PNN, BCAN, and NCAN IR	Barnes maze reversal learning	Normal adolescent performance, ↓ adult performance	Coleman et al., 2014
Adolescent intermittent alcohol exposure	Rat	Striatum, OFC, and mPFC	26–30 days	↑ PNN number and PNN apposing PVB	NA	NA	Dannenhoffer et al., 2022
DID (6 weeks)	Mouse	Insula	20 h	↑ PNN intensity, ACAN, BCAN, and PCAN	NA	NA	Chen et al., 2015
Two-bottle choice: quinine-alcohol (4 days)	Mouse	Insula	NA	NA	Intra-insula chABC injection before two-bottle choice	↑ Aversion to quinine-adulterated alcohol	Chen and Lasek, 2020
Alcohol use disorder	Human	Blood	GWAS	↑ Frequency of -1562C/T polymorphism on MMP-9	NA	NA	Samochowiec et al., 2010
Alcohol use disorder	Human	Blood	GWAS	Single nucleotide polymorphisms on TNN and TNR genes	NA	NA	Zuo et al., 2012

Acute (top) and chronic (bottom) effects of alcohol on ECM molecules. Preharvest interval denotes the time between the most recent drug exposure and euthanasia. SA, self-administration; CPP, conditioned place preference; DID, 4-day drinking in the dark; HPC, hippocampus; mPFC, medial prefrontal cortex; OFC, orbitofrontal cortex; MMP, matrix metalloproteinase; PNN, perineuronal net; ACAN, aggrecan; BCAN, brevican; PCAN, phosphacan; chABC, chondroitinase ABC; TNN, tenascin-N; TNR, tenascin-R; IR, immunoreactivity.

#### Acute alcohol exposure

One of the first studies to examine brain levels of ECM molecules in response to drugs of abuse focused on alcohol’s effect on MMP-9 in the rat brain during water maze training (Wright et al., 2003). Alcohol administration resulted in reduced MMP-9 activity in the hippocampus and prefrontal cortex. This reduction was evident after 2 days and became progressively more severe after 4 and 6 days of alcohol administration (Wright et al., 2003). The progressive decrease in MMP-9 activity was accompanied by impaired water maze performance, suggesting that alcohol impairs hippocampal learning by downregulating MMP-9 activity (Wright et al., 2003). Furthermore, intracerebroventricular administration of the broad spectrum MMP inhibitor, FN-439, during ethanol withdrawal, was reported to prevent relapse to alcohol self-administration in rats (Smith et al., 2011). Alternatively, a recent study using chronic adenovirus-mediated overexpression of MMP-9 in the hippocampus demonstrated that mice with chronic MMP-9 overexpression had reduced alcohol-induced place preference (Yin et al., 2023). Persistent MMP-9 upregulation also perturbed ethanol-induced increases in NMDA receptor subtypes (Yin et al., 2023). Inhibitory effects of prolonged upregulation of MMP-9 on alcohol place preference points to the importance of temporal specificity of MMP regulation during phases of reward integration. Upregulation of MMPs, when prolonged, may cause aberrant synaptogenesis that inhibits the normal synaptic refinement and GABAergic (i.e., parvalbumin) modulatory processes which aid in reward memory precision and consolidation (Beroun et al., 2019).

While no studies have examined PNNs after acute exposure to alcohol (i.e., <24 h), exposure to alcohol for one week does not significantly alter the fluorescent intensity of PNNs, or mRNA expression of the PNN components aggrecan, brevican, and phosphacan (Chen et al., 2015). Lack of changes in CSPG levels at this timepoint may indicate that ECM remodeling occurs earlier than 2 days, and at 7 days ECM remodeling remains in an interim phase where neither MMPs nor PNNs/their components are significantly altered.

#### Chronic alcohol use

Several studies have investigated how chronic alcohol exposure regulates the structure of the ECM. Adolescent exposure to alcohol in mice results in increased densities of PNNs and increased immunoreactivity of brevican and neurocan in the orbitofrontal cortex (OFC) that is sustained into adulthood (Coleman et al., 2014; Dannenhoffer et al., 2022). In adult rats, 6 weeks of alcohol self-administration significantly increases the intensity of WFA-labeled PNNs and mRNA levels of aggrecan, brevican, and phosphacan in the insula (Chen et al., 2015). A set of experiments from the same group used cocktails of quinine, an aversive and bitter additive, with alcohol to investigate whether the ECM regulates aversion-resistant drinking (Chen and Lasek, 2020). Ablating PNNs in the insula with chABC 3 days prior to the onset of alcohol self-administration rendered animals more sensitive to aversive quinine cocktails (Chen and Lasek, 2020). As the insula is part of the gustatory cortex, increased PNNs in this area may contribute to integration of the non-pharmacologically relevant aspects of alcohol into the memory trace (i.e., bitter taste). In comparison, the ECM neural cell adhesion molecules (NrCAMs) may be involved in the contextual aspects of alcohol reward circuits. NrCAM knockout mice display reduced alcohol place preference (Ishiguro et al., 2014), and pharmacological inhibition of this signaling pathway via systemic administration of prolyl-leucyl-glycinamide also impaired alcohol place preference, suggesting a potential ECM-based therapeutic approach for alleviating context-induced relapse. Chronic alcohol consumption is also associated with increased neuroinflammation, such as heightened recruitment of several pro-inflammatory cytokines that are known to impact the ECM (Kelley and Dantzer, 2011; Lowe et al., 2020; Xiong et al., 2022). Genetic studies of AUD identified associations of genes encoding ECM molecules with increased risk of AUD. Two genome-wide association studies implicate genetic polymorphisms of several ECM genes including tenascin-N and -R and MMP-9 with AUD (Samochowiec et al., 2010; Zuo et al., 2012), suggesting that ECM molecules may be involved in the susceptibility for developing AUD.

### Psychoplastogens

Psychoplastogens encompass a variety of chemical classes (e.g., dissociatives, psychedelics) which produce profound, long-lasting effects on neural plasticity with single exposures (Ly et al., 2018; Ballentine et al., 2022). This feature of psychoplastogens has made them attractive candidates as potential treatments for SUD and MDD in controlled, clinical settings in combination with psychotherapy, such as cognitive behavioral therapy (CBT) (Carhart-Harris et al., 2012; Talin and Sanabria, 2017; Rieser et al., 2022; Sloshower et al., 2023).

Ketamine, an NMDA receptor antagonist originally used as an anesthetic, has psychotomimetic effects including hallucinations (Powers et al., 2015). It is also used recreationally and can lead to SUD (Strous et al., 2022). Chronic recreational use reportedly results in structural gray and white matter alterations and memory impairment (Strous et al., 2022). Several studies, including clinical trials, provide support for the use of low dose ketamine administration for treatment-resistant depression (Zarate et al., 2006; Phillips et al., 2019; Can et al., 2021), which is often comorbid with SUD (Shin and Kim, 2020). Repeated exposure to ketamine at anesthetic doses results in decreased composition of PNNs via microglia-mediated proteolytic ECM degradation which promotes synapse plasticity (Venturino and Siegert, 2021; Venturino et al., 2021). Further, ketamine and phencyclidine (PCP) administration alter expression of a number of ECM genes, including the collagen type IX alpha 2 chain, NrCAM-1, decorin, and heparan sulfate 6-O-sulfotransferase, in the striatum between one and 8 h following administration (Oommen et al., 2023), suggesting that effects on the ECM are much broader than reported PNN composition changes.

The conventional serotonergic psychedelics, lysergic acid diethylamide (LSD), *N,N*-dimethyltryptamine (DMT), and psilocybin, are suggested to have potentially rapid antidepressant effects with a relatively limited side effect profile (Carhart-Harris et al., 2016; Rucker et al., 2016). In the rat PFC, both *in vivo* and *in vitro*, LSD, DMT, and the substituted amphetamine psychedelic 2,5-dimethoxy-4-iodoamphetamine (DOI) promote neuritogenesis, spinogenesis and synaptogenesis (Ly et al., 2018). Furthermore, the synaptic changes induced by psychedelics may be long term. For example, the reported increase in dendritic remodeling detected within 24 h was reported to last for at least 1 month following a single dose of psilocybin (Shao et al., 2021). To date, there are no studies of how these compounds regulate the ECM. Acute administration of DOI produces robust transcriptional responses by recruiting multiple cell types including glia, and significantly increases expression of parvalbumin and somatostatin in activated neuronal ensembles (Martin and Nichols, 2016). In line with this, both psilocybin and ketamine upregulate parvalbumin expression in neurons expressing the immediate early gene cFos (Davoudian et al., 2023). Upregulated somatostatin, a neurotransmitter expressed by a subset of interneurons ensheathed by PNNs (Willis et al., 2022), may potentially alleviate molecular alterations in the case of SUD, as our recent study identified decreased hippocampal somatostatin expression in subjects with SUD (Valeri et al., 2022). Taken together, we speculate that psychedelic drugs may have similar actions on the ECM as psychostimulant drugs, mediating rapid and sustained ECM disassembly upon administration. However, serotonergic psychedelics are not reinforcing, and therefore likely do not carry risk of addiction (Nichols, 2016). Several studies suggest that psychedelics combined with CBT may be promising candidates for improving SUD outcomes and preventing relapse, as they may promote plasticity of brain circuits involved in reward and goal-directed behavior (DiVito and Leger, 2020), potentially in part through ECM remodeling.

### Obesity and metabolic disorders

Obesity is a global health problem that impacts virtually all aspects of health and results in a significant personal and societal burden (Main et al., 2010). Obesity also increases the risk of a range of diseases, including metabolic disorders, which encompasses cardiovascular disease, diabetes mellitus, and hypertension (Colditz et al., 1995; Calle et al., 2003; GBD 2015 Obesity Collaborators et al., 2017). Several psychiatric disorders associated with increased risk of SUD share comorbidity with metabolic disorders (Luppino et al., 2010; Jimenez et al., 2019; Trevino-Alvarez et al., 2023). For example, MDD is often comorbid with type 2 diabetes (T2D), and several lines of evidence point to a core metabolic pathology across mood disorders (Fagiolini et al., 2002; Dona et al., 2020; Norwitz et al., 2020). Additionally, conditions associated with metabolic disorders such as painful diabetic neuropathy are commonly managed with opioid medications, which pose a risk of addiction (Callaghan et al., 2012; Jensen et al., 2021). In addition, many people with T2D (over 40%) also have a comorbid SUD (Wu et al., 2018; Feldman et al., 2019). The brain regions primarily responsible for food-seeking behaviors include the hypothalamus (e.g., arcuate nucleus, median eminence, lateral hypothalamus), hippocampus (Martin and Davidson, 2014; Parent et al., 2014), and PFC (Garcia-Garcia et al., 2014). Obesogenic diets have been reported to result in inflammation and alterations in synaptic plasticity and blood-brain barrier regulation, all of which are regulated in part by the ECM (Gustafson et al., 2007; Guillemot-Legris and Muccioli, 2017; Matikainen-Ankney and Kravitz, 2018; Brown and Sorg, 2023).

Several studies support a key role of PNNs in the arcuate nucleus in obesity and metabolic disorders (Table 5). Formation of PNNs in the arcuate nucleus coincides with the closure of the critical period of agouti-related peptide neuron maturation in this region, in a leptin-dependent manner (Mirzadeh et al., 2019). Furthermore, Zucker diabetic fatty rats, which carry spontaneous missense mutations of the leptin receptor gene causing hyperglycemia and rapid development of T2D, have significantly reduced PNNs and altered sulfation of CSPGs in the arcuate nucleus (Alonge et al., 2020). These effects on PNNs are reversed by intracerebroventricular injections of fibroblast growth factor 1, which induces sustained diabetes remission in Zucker rats (Scarlett et al., 2016; Alonge et al., 2020).

**TABLE 5:** Involvement of the ECM in diet and metabolism.

Manipulation	Species	Brain area	ECM response	Treatment	Response	References
Leptin deficiency (ob/ob)	Mouse	Arcuate nucleus (ARC)	↓ PNN apposition on AgRP	Leptin supplement	Rescued PNN abundance	Mirzadeh et al., 2019
ZDF and high-fat diet	Rat	ARC	↓ PNNs	Fibroblast growth factor-1 i.c.v. injection	Rescued PNN abundance and sulfation	Alonge et al., 2020
			Altered CS/DS sulfation			
HFD	Rat	PL-PFC	↓ PNN intensity, but not number	NA	NA	Dingess et al., 2018
		IL-PFC	No changes			
		OFC	↓ PNN intensity and number			
Obesity-prone male rats (normal diet vs. HFD)	Rat	PL-PFC	↓ PNN intensity and PVB apposition	NA	NA	Dingess et al., 2020
		IL-PFC	No changes			
		OFC	↓ PNN intensity number, and PVB apposition			
Obesity-prone female rats (normal diet vs. HFD)	Rat	PL-PFC	No changes	NA	NA	Dingess et al., 2020
		IL-PFC	↑ PNN intensity, number and PVB apposition			
		OFC	↓ PNN intensity			
Obesity-resistant male rats (normal diet vs. HFD)	Rat	PL-PFC	No changes	NA	NA	Dingess et al., 2020
		IL-PFC	↓ PNN intensity			
		OFC	No changes			
Obesity-resistant female rats (normal diet vs. HFD)	Rat	PL-PFC	No changes	NA	NA	Dingess et al., 2020
		IL-PFC	↓ PNN intensity			
		OFC	No changes			
HF-HSD	Mouse	PL-PFC	↑ active microglia	NA	NA	Reichelt et al., 2021
		IL-PFC	↑ active microglia			
		OFC	↑ active microglia			
		HPC	↓ PNNs and ↑ active microglia			
Sucrose self-administration	Rat	PL-, IL-PFC, and OFC	No changes	NA	NA	Slaker et al., 2016b
Overnight fast and 1 h re-feed (ob/ob)	Mouse	ARC and ME	↑ PNN intensity in ME following re-feed	MBH chABC injection	↓ Food intake 48 – 96 h post chABC	Kohnke et al., 2021
Male HFD	Mouse	ARC and TE	No changes in TE	Castration	↓ PNN intensity in ARC	Zhang et al., 2021
Female HFD	Mouse	ARC and TE	↑ PNN intensity in TE	Ovariectomy	↓ PNN intensity in ARC	Zhang et al., 2021

Acute (top) and chronic (bottom) effects of cocaine, methamphetamine, and nicotine on ECM molecules. Preharvest interval denotes the time between the most recent drug exposure and euthanasia. ZDF, Zucker diabetic fatty rat; HFD, high fat diet; HF-HSD, high fat-high sugar diet; OP, obesity-prone; OR, obesity-resistant; ARC, arcuate nucleus; ME, median eminence; TE, terete hypothalamic nucleus; HPC, hippocampus; PFC, prefrontal cortex; IL, infralimbic; PL, prelimbic; OFC, orbitofrontal cortex; PNN, perineuronal net; AgRP, agouti-related protein; PVB, parvalbumin; CS, chondroitin sulfate; DS, dermatan sulfate.

Several studies also report ECM alterations in several brain areas outside of the hypothalamus in preclinical models of obesity (Table 5). For example, chronic (3 weeks) exposure to a high-diet in adult male rats decreased the fluorescent intensity of PNNs labeled with WFA in the prelimbic and orbitofrontal cortices, an effect that was not contingent on weight gain (Dingess et al., 2018). In a report from the same group, Sprague-Dawley rats which were bred either prone to obesity or resistant to obesity had differential responses a high-fat diet in the OFC, with decreases in PNN intensity in obese-prone rats and increased PNN intensity in obese-resistant rats (Dingess et al., 2020). Chronic exposure (5 weeks) to a high-fat/high-sugar diet decreased PNN density in the CA1 field of the hippocampus of adult male mice, along with corresponding increases in adiposity and abundance of activated microglia (Reichelt et al., 2021). However, no changes were observed in PNNs in the PFC in the same animals (Reichelt et al., 2021). Although high-fat/high-sugar diet formulations induce changes in PNNs in the PFC, sugar alone does not appear to have any effect on PNNs (Slaker et al., 2016b; Roura-Martinez et al., 2020). Data regarding short term effects of feeding and specific diets on the ECM is greatly limited. A recent study suggests that PNN composition in the median eminence of the hypothalamus is decreased during fasting but PNNs increase after a brief feeding following the fasting period (Kohnke et al., 2021).

A relatively limited set of studies have investigated sexual dimorphic effects of diet on the ECM. In contrast to the data in males, both outbred and obese-prone female rats exhibit an increase in PNN intensity in the infralimbic cortex following a high fat diet, whereas obese-resistant females show a decrease in PNN intensity (Dingess et al., 2020). In contrast, in the arcuate nucleus, sex hormones have been shown to affect the ECM independent of dietary manipulation (Zhang et al., 2021).

Obesity as well as T2D contribute to neuroinflammation (Miller and Spencer, 2014; Van Dyken and Lacoste, 2018). Brain-wide decreases of PNNs in male subjects exposed to a chronic high-fat diet is in line with heightened inflammatory activity from microglia and potential proteolysis of ECM. There is likely deep complexity between multiple factors that correspond with diet-induced differences in the architecture of the ECM, such as genetic predisposition, age, and sex. Future studies examining these relationships and how they interact in comorbid conditions will provide key insight into the involvement of the ECM in metabolic disorders and how this information can be leveraged for therapeutic strategies. As mentioned previously, metabolic dysfunction is a characteristic of some psychiatric disorders, such as MDD and BD. The ketogenic (low carb) diet is rapidly gaining support as a treatment for psychiatric disorders including mood disorders, autism spectrum disorder, and schizophrenia (Brietzke et al., 2018; Campbell and Campbell, 2020; Sarnyai and Palmer, 2020; Danan et al., 2022). Intriguingly, the ketogenic diet has been shown to alter the ECM by downregulating gene expression of fibrinogen, but upregulating myelin basic protein expression in the rat hippocampus (Noh et al., 2004).

## Current challenges and future directions

Despite the growing evidence for a key role of ECM molecules in substance use disorders, there are several knowledge gaps that limit the development of ECM-based pharmacotherapies. First, as indicated in this review, despite the growing number of studies, there is a lack of sufficient information regarding the temporal ECM changes in specific brain regions for specific substances. In addition, despite evidence for WFA labeled PNN alterations, there is a lack of information regarding changes in PNN composition and specific ECM structures such as perisynaptic ECM, axonal coats and/or perivascular ECM. Moreover, few studies thus far have examined the role of the ECM in neurodevelopmental factors prior to the onset of SUD that can contribute to risk of SUD, including genetic factors, chronic stress, exposure to a culture of drug use, traumatic experiences, and socio-economic status. Future studies on the ECM that consider such etiological factors would provide critical insight into the role of the ECM in enhanced risk to SUDs.

Furthermore, the vast majority of current evidence is largely based on animal model studies, there is currently a lack of information regarding these ECM structures in the brain of individuals with SUD. Human postmortem studies may provide critical information that can support preclinical studies and guide development of therapeutic strategies. Polysubstance use disorder is the most common clinical presentation of SUD, thus preclinical studies of polysubstance use may provide key insight into potential interactions of various drugs of abuse on the ECM and allow for development of more translatable therapeutic strategies. In terms of developing or repurposing drugs for ECM based treatments of SUD, the current evidence suggests that MMP inhibitors such as doxycycline may inhibit ECM degradation and formation of reward memories associated with initial drug exposure. In comparison, short-term upregulation of MMPs may represent an effective strategy to treat chronic SUD, especially in combination with CBT. Temporary removal of PNNs may allow for reorganization or weakening of reward associated synapses, and may prevent feedforward modulation of neuronal ensembles by parvalbumin, thus creating more glutamatergic noise and less coherence of the neurons that are associated with drug memories (Brown and Sorg, 2023). Furthermore, several lines of evidence indicate that inhibiting MMP activity may be useful in preventing relapse following a period of abstinence. The development of pharmacotherapies with selectivity for brain ECM structures which can be delivered systemically, provide temporal control, and limit potential off-target side effects remains a major challenge for ECM-based therapeutic strategies.

Regarding the role of the ECM in metabolic disorders, a limitation of rodent models of T2D (e.g., the Zucker rat) is the relatively abrupt development of hyperglycemia, which is not characteristic of the gradual human disease progression (Oltman et al., 2005; Hinder et al., 2018). As rodent models of T2D evolve, examining the role of the ECM in the progression from prediabetic-like states to T2D may provide evidence for temporal ECM alterations that can guide preventative and treatment strategies. As previously mentioned, many individuals with mood disorders often suffer from metabolic disorders and eating disorders, such as binge-eating disorder. In this context, there is currently a lack of evidence providing a link between mood dysregulation, HFD, and the ECM. Studies focused on these associations may enhance our understanding of potential ECM-based precipitating factors for metabolic disorders.

## Concluding remarks

Current evidence suggests that the ECM is at the intersection of synaptic regulation and neuroimmune responses to drug reward and food reward. Synaptic regulation and inflammation are hallmarks of the neuroadaptations induced by both SUD and metabolic disorders (Cui et al., 2014; Hao et al., 2016). These processes may impact overlapping neuroanatomical circuits and cellular mechanisms. Food is essential for survival, and evolutionarily conserved processes may promote strengthening of brain circuits involved in integrating environmental cues associated with biologically relevant rewards such as food, especially food that is high in fat and calories. In comparison, drugs of abuse are not necessary for survival, and initial exposure to drugs most commonly occurs after the closure of critical periods of plasticity associated with maturation of PNNs. Therefore, enhanced PNNs by chronic drug (unnatural) reward may be the result of synaptic reorganization occurring later in development, recruiting additional ECM components to stabilize synapses that participate in integration of novel, potent reward experiences engaging brain circuits that are normally involved in regulating memories for experiences that are necessary for survival. Intriguingly, a similar temporal relationship of PNN alterations was reported in the rat hippocampus across stages of social defeat stress (Riga et al., 2017), suggesting that this temporal regulation of PNNs may be a shared mechanism underlying memory processing across positive and negative experiences. Decreased PNNs in obesity and metabolic disorders may instead reflect increased neuroinflammation associated with these conditions.

**FIGURE 3:**
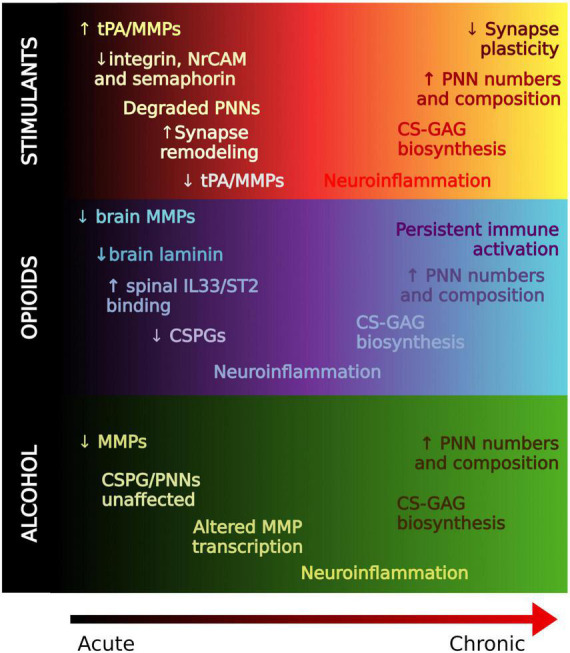
Regulation of the extracellular matrix in drug memory processing. A working hypothesis of ECM alterations from acute to chronic stages of substance use. In general, ECM molecules are degraded in acute stages, possibly to allow for formation of new synapses. PNNs and ECM molecules are generally increased with chronic use which may contribute to the strength of reward memories associated with SUD that confer relapse.

In summary, ECM molecules may represent key contributors to the pathogenesis of SUD and metabolic disorders. Acute exposure to substances of abuse render PNNs unstable through the activity of endogenous proteases, and chronic exposure generally increases PNNs and ECM molecules (Figure 3). Pathologically, SUD is an ingrained memory trace that maladaptively triggers activation of brain circuits that recurrently promote relapse. Thus, enhanced PNNs may reflect an inflexibility of memory systems pertaining to reward-associated stimuli which perpetuates the cycle of relapse and addiction.

## Author contributions

All authors listed have made a substantial, direct, and intellectual contribution to the work, and approved it for publication.

## References

[B1] Adams I. Brauer K. Arelin C. Hartig W. Fine A. Mader M. (2001). Perineuronal nets in the rhesus monkey and human basal forebrain including basal ganglia. *Neuroscience* 108 285–298. 10.1016/s0306-4522(01)00419-5 11734361

[B2] Ajmo J. Eakin A. Hamel M. Gottschall P. (2008). Discordant localization of WFA reactivity and brevican/ADAMTS-derived fragment in rodent brain. *BMC Neurosci.* 9:14. 10.1186/1471-2202-9-14 18221525 PMC2263047

[B3] Alaiyed S. McCann M. Mahajan G. Rajkowska G. Stockmeier C. Kellar K. (2020). Venlafaxine stimulates an MMP-9-dependent increase in excitatory/inhibitory balance in a stress model of depression. *J. Neurosci.* 40 4418–4431. 10.1523/JNEUROSCI.2387-19.2020 32269106 PMC7252486

[B4] Alonge K. Logsdon A. Murphree T. Banks W. Keene C. Edgar J. (2019). Quantitative analysis of chondroitin sulfate disaccharides from human and rodent fixed brain tissue by electrospray ionization-tandem mass spectrometry. *Glycobiology* 29 847–860. 10.1093/glycob/cwz060 31361007 PMC6861844

[B5] Alonge K. Mirzadeh Z. Scarlett J. Logsdon A. Brown J. Cabrales E. (2020). Hypothalamic perineuronal net assembly is required for sustained diabetes remission induced by fibroblast growth factor 1 in rats. *Nat. Metab.* 2 1025–1033. 10.1038/s42255-020-00275-6 32895577 PMC7572652

[B6] Alsio J. Olszewski P. Levine A. Schioth H. (2012). Feed-forward mechanisms: addiction-like behavioral and molecular adaptations in overeating. *Front. Neuroendocrinol.* 33:127–139. 10.1016/j.yfrne.2012.01.002 22305720

[B7] American Psychiatric Association (2013). *Diagnostic and statistical manual of mental disorders, (DSM-5)*, Fifth Edn. Virginia: American Psychiatric Publishing, Inc.

[B8] Asher R. Morgenstern D. Moon L. Fawcett J. (2001). Chondroitin sulphate proteoglycans: inhibitory components of the glial scar. *Prog. Brain Res.* 132 611–619. 10.1016/S0079-6123(01)32106-4 11545024

[B9] Baeten K. Akassoglou K. (2011). Extracellular matrix and matrix receptors in blood-brain barrier formation and stroke. *Dev. Neurobiol.* 71 1018–1039. 21780303 10.1002/dneu.20954 PMC3482610

[B10] Bahi A. Dreyer J. (2005). Cocaine-induced expression changes of axon guidance molecules in the adult rat brain. *Mol. Cell Neurosci.* 28 275–291. 10.1016/j.mcn.2004.09.011 15691709

[B11] Bahi A. Kusnecov A. Dreyer J. (2008). Effects of urokinase-type plasminogen activator in the acquisition, expression and reinstatement of cocaine-induced conditioned-place preference. *Behav. Brain Res.* 191 17–25. 10.1016/j.bbr.2008.03.004 18436315

[B12] Baik J. (2013). Dopamine signaling in food addiction: role of dopamine D2 receptors. *BMB Rep.* 46 519–526. 10.5483/bmbrep.2013.46.11.207 24238362 PMC4133846

[B13] Ballentine G. Friedman S. Bzdok D. (2022). Trips and neurotransmitters: discovering principled patterns across 6850 hallucinogenic experiences. *Sci. Adv.* 8:eabl6989. 10.1126/sciadv.abl6989 35294242 PMC8926331

[B14] Barry D. Clarke M. Petry N. (2009). Obesity and its relationship to addictions: is overeating a form of addictive behavior? *Am. J. Addict.* 18 439–451. 10.3109/10550490903205579 19874165 PMC2910406

[B15] Bekku Y. Rauch U. Ninomiya Y. Oohashi T. (2009). Brevican distinctively assembles extracellular components at the large diameter nodes of Ranvier in the CNS. *J. Neurochem.* 108 1266–1276. 10.1111/j.1471-4159.2009.05873.x 19141078

[B16] Beroun A. Mitra S. Michaluk P. Pijet B. Stefaniuk M. Kaczmarek L. (2019). MMPs in learning and memory and neuropsychiatric disorders. *Cell Mol. Life Sci.* 76 3207–3228. 10.1007/s00018-019-03180-8 31172215 PMC6647627

[B17] Blacktop J. Todd R. Sorg B. (2017). Role of perineuronal nets in the anterior dorsal lateral hypothalamic area in the acquisition of cocaine-induced conditioned place preference and self-administration. *Neuropharmacology* 118 124–136. 10.1016/j.neuropharm.2017.03.018 28322980 PMC5492967

[B18] Blanco I. Conant K. (2021). Extracellular matrix remodeling with stress and depression: studies in human, rodent and zebrafish models. *Eur. J. Neurosci.* 53 3879–3888. 10.1111/ejn.14910 32673433 PMC7967247

[B19] Blum K. Werner T. Carnes S. Carnes P. Bowirrat A. Giordano J. (2012). Sex, drugs, and rock ‘n’ roll: hypothesizing common mesolimbic activation as a function of reward gene polymorphisms. *J. Psychoactive Drugs.* 44 38–55. 10.1080/02791072.2012.662112 22641964 PMC4040958

[B20] Brew K. Dinakarpandian D. Nagase H. (2000). Tissue inhibitors of metalloproteinases: evolution, structure and function. *Biochim. Biophys. Acta* 1477 267–283. 10.1016/s0167-4838(99)00279-4 10708863

[B21] Brietzke E. Mansur R. Subramaniapillai M. Balanzá-Martínez V. Vinberg M. González-Pinto A. (2018). Ketogenic diet as a metabolic therapy for mood disorders: evidence and developments. *Neurosci. Biobehav. Rev.* 94 11–16. 10.1016/j.neubiorev.2018.07.020 30075165

[B22] Brown T. Sorg B. (2023). Net gain and loss: influence of natural rewards and drugs of abuse on perineuronal nets. *Neuropsychopharmacology* 48 3–20. 10.1038/s41386-022-01337-x 35568740 PMC9700711

[B23] Brown T. Forquer M. Cocking D. Jansen H. Harding J. Sorg B. (2007). Role of matrix metalloproteinases in the acquisition and reconsolidation of cocaine-induced conditioned place preference. *Learn. Mem.* 14 214–223. 10.1101/lm.476207 17353546 PMC1838561

[B24] Brown T. Forquer M. Harding J. Wright J. Sorg B. (2008). Increase in matrix metalloproteinase-9 levels in the rat medial prefrontal cortex after cocaine reinstatement of conditioned place preference. *Synapse* 62 886–889. 10.1002/syn.20562 18792988

[B25] Brown T. Wilson A. Cocking D. Sorg B. (2009). Inhibition of matrix metalloproteinase activity disrupts reconsolidation but not consolidation of a fear memory. *Neurobiol. Learn. Mem.* 91 66–72. 10.1016/j.nlm.2008.09.003 18824238 PMC2719776

[B26] Browne C. Futamura R. Minier-Toribio A. Hicks E. Ramakrishnan A. Martínez-Rivera F. (2023). Transcriptional signatures of heroin intake and seeking throughout the brain reward circuit. *bioRxiv [Preprint]* 10.1101/2023.01.11.523688 37294757 PMC10256172

[B27] Bruckner G. Morawski M. Arendt T. (2008). Aggrecan-based extracellular matrix is an integral part of the human basal ganglia circuit. *Neuroscience* 151 489–504. 10.1016/j.neuroscience.2007.10.033 18055126

[B28] Brzdak P. Nowak D. Wiera G. Mozrzymas J. (2017). Multifaceted roles of metzincins in CNS physiology and pathology: from synaptic plasticity and cognition to neurodegenerative disorders. *Front. Cell. Neurosci.* 11:178. 10.3389/fncel.2017.00178 28713245 PMC5491558

[B29] Cabungcal J. Steullet P. Morishita H. Kraftsik R. Cuenod M. Hensch T. (2013). Perineuronal nets protect fast-spiking interneurons against oxidative stress. *Proc. Natl. Acad. Sci. U.S.A.* 110 9130–9135. 10.1073/pnas.1300454110 23671099 PMC3670388

[B30] Callaghan B. Cheng H. Stables C. Smith A. Feldman E. (2012). Diabetic neuropathy: clinical manifestations and current treatments. *Lancet Neurol.* 11 521–534. 10.1016/S1474-4422(12)70065-0 22608666 PMC4254767

[B31] Calle E. Rodriguez C. Walker-Thurmond K. Thun M. (2003). Overweight, obesity, and mortality from cancer in a prospectively studied cohort of U.S. adults. *N. Engl. J. Med.* 348 1625–1638. 10.1056/NEJMoa021423 12711737

[B32] Campbell I. Campbell H. (2020). Mechanisms of insulin resistance, mitochondrial dysfunction and the action of the ketogenic diet in bipolar disorder. Focus on the PI3K/AKT/HIF1-a pathway. *Med. Hypotheses* 145:110299. 10.1016/j.mehy.2020.110299 33091780

[B33] Can A. Hermens D. Dutton M. Gallay C. Jensen E. Jones M. (2021). Low dose oral ketamine treatment in chronic suicidality: an open-label pilot study. *Transl. Psychiatry* 11:101. 10.1038/s41398-021-01230-z 33542187 PMC7862447

[B34] Carhart-Harris R. Bolstridge M. Rucker J. Day C. Erritzoe D. Kaelen M. (2016). Psilocybin with psychological support for treatment-resistant depression: an open-label feasibility study. *Lancet Psychiatry* 3 619–627. 10.1016/S2215-0366(16)30065-7 27210031

[B35] Carhart-Harris R. Leech R. Williams T. Erritzoe D. Abbasi N. Bargiotas T. (2012). Implications for psychedelic-assisted psychotherapy: functional magnetic resonance imaging study with psilocybin. *Br. J. Psychiatry* 200 238–244. 10.1192/bjp.bp.111.103309 22282432

[B36] Carter B. Tiffany S. (1999). Meta-analysis of cue-reactivity in addiction research. *Addiction* 94 327–340. 10605857

[B37] Carulli D. Verhaagen J. (2021). An extracellular perspective on CNS maturation: perineuronal nets and the control of plasticity. *Int. J. Mol. Sci.* 22:2434. 10.3390/ijms22052434 33670945 PMC7957817

[B38] Chen H. Lasek A. (2020). Perineuronal nets in the insula regulate aversion-resistant alcohol drinking. *Addict. Biol.* 25:e12821. 10.1111/adb.12821 31433552 PMC7032993

[B39] Chen H. He D. Lasek A. (2015). Repeated binge drinking increases perineuronal nets in the insular cortex. *Alcohol Clin. Exp. Res.* 39 1930–1938. 10.1111/acer.12847 26332441 PMC4592458

[B40] Chen J. Kawamura T. Sethi M. Zaia J. Repunte-Canonigo V. Sanna P. (2017). Heparan sulfate: resilience factor and therapeutic target for cocaine abuse. *Sci. Rep.* 7:13931. 10.1038/s41598-017-13960-6 29066725 PMC5654972

[B41] Chen J. Repunte-Canonigo V. Kawamura T. Lefebvre C. Shin W. Howell L. (2013). Hypothalamic proteoglycan syndecan-3 is a novel cocaine addiction resilience factor. *Nat. Commun.* 4:1955. 10.1038/ncomms2955 23736082 PMC3709481

[B42] Chioma V. Kruyer A. Bobadilla A. Angelis A. Ellison Z. Hodebourg R. (2021). Heroin seeking and extinction from seeking activate matrix metalloproteinases at synapses on distinct subpopulations of accumbens cells. *Biol. Psychiatry* 89 947–958. 10.1016/j.biopsych.2020.12.004 33579535 PMC8434769

[B43] Colditz G. Willett W. Rotnitzky A. Manson J. (1995). Weight gain as a risk factor for clinical diabetes mellitus in women. *Ann. Intern. Med.* 122 481–486. 10.7326/0003-4819-122-7-199504010-00001 7872581

[B44] Coleman L. Jr. Liu W. Oguz I. Styner M. Crews F. (2014). Adolescent binge ethanol treatment alters adult brain regional volumes, cortical extracellular matrix protein and behavioral flexibility. *Pharmacol. Biochem. Behav.* 116 142–151. 10.1016/j.pbb.2013.11.021 24275185 PMC3913047

[B45] Cui C. Shurtleff D. Harris R. (2014). Neuroimmune mechanisms of alcohol and drug addiction. *Int. Rev. Neurobiol.* 118 1–12. 10.1016/b978-0-12-801284-0.00001-4 25175859 PMC4804710

[B46] Danan A. Westman E. Saslow L. Ede G. (2022). The ketogenic diet for refractory mental illness: a retrospective analysis of 31 inpatients. *Front. Psychiatry.* 13:951376. 10.3389/fpsyt.2022.951376 35873236 PMC9299263

[B47] Dankovich T. Rizzoli S. (2022). Extracellular matrix recycling as a novel plasticity mechanism with a potential role in disease. *Front. Cell. Neurosci.* 16:854897. 10.3389/fncel.2022.854897 35431813 PMC9008140

[B48] Dannenhoffer C. Gomez A. Macht V. Jawad R. Sutherland E. Vetreno R. (2022). Impact of adolescent intermittent ethanol exposure on interneurons and their surrounding perineuronal nets in adulthood. *Alcohol Clin. Exp. Res.* 46 759–769. 10.1111/acer.14810 35307830 PMC9117471

[B49] Dauth S. Grevesse T. Pantazopoulos H. Campbell P. Maoz B. Berretta S. (2016). Extracellular matrix protein expression is brain region dependent. *J. Comp. Neurol.* 524 1309–1336. 10.1002/cne.23965 26780384 PMC7714387

[B50] Davoudian P. Shao L. Kwan A. (2023). Shared and distinct brain regions targeted for immediate early gene expression by ketamine and psilocybin. *ACS Chem. Neurosci.* 14 468–480. 10.1021/acschemneuro.2c00637 36630309 PMC9898239

[B51] de Guglielmo G. Iemolo A. Nur A. Turner A. Montilla-Perez P. Martinez A. (2022). Reelin deficiency exacerbates cocaine-induced hyperlocomotion by enhancing neuronal activity in the dorsomedial striatum. *Genes Brain Behav.* 21:e12828. 10.1111/gbb.12828 35906757 PMC9744517

[B52] de Winter F. Kwok J. Fawcett J. Vo T. Carulli D. Verhaagen J. (2016). The chemorepulsive protein semaphorin 3A and perineuronal net-mediated plasticity. *Neural Plasticity* 2016:3679545. 10.1155/2016/3679545 27057361 PMC4738953

[B53] Deepa S. Carulli D. Galtrey C. Rhodes K. Fukuda J. Mikami T. (2006). Composition of perineuronal net extracellular matrix in rat brain: a different disaccharide composition for the net-associated proteoglycans. *J. Biol. Chem.* 281 17789–17800. 10.1074/jbc.M600544200 16644727

[B54] Dick G. Tan C. Alves J. Ehlert E. Miller G. Hsieh-Wilson L. (2013). Semaphorin 3A binds to the perineuronal nets via chondroitin sulfate type E motifs in rodent brains. *J. Biol. Chem.* 288 27384–27395. 10.1074/jbc.M111.310029 23940048 PMC3779733

[B55] Dingess P. Harkness J. Slaker M. Zhang Z. Wulff S. Sorg B. (2018). Consumption of a high-fat diet alters perineuronal nets in the prefrontal cortex. *Neural Plast.* 2018:2108373. 10.1155/2018/2108373 29849552 PMC5937429

[B56] Dingess P. Zhang Z. Sorg B. Ferrario C. Brown T. (2020). Sex and region-specific effects of high fat diet on PNNs in obesity susceptible rats. *Physiol. Behav.* 222:112963. 10.1016/j.physbeh.2020.112963 32416158 PMC8249905

[B57] Dityatev A. Schachner M. Sonderegger P. (2010). The dual role of the extracellular matrix in synaptic plasticity and homeostasis. *Nat. Rev. Neurosci.* 11 735–746. 10.1038/nrn2898 20944663

[B58] DiVito A. Leger R. (2020). Psychedelics as an emerging novel intervention in the treatment of substance use disorder: a review. *Mol. Biol. Rep.* 47 9791–9799. 10.1007/s11033-020-06009-x 33231817

[B59] Dona A. DeLouize A. Eick G. Thiele E. Salinas Rodriguez A. Manrique Espinoza B. (2020). Inflammation and central adiposity as mediators of depression and uncontrolled diabetes in the study on global AGEing and adult health (SAGE). *Am. J. Hum. Biol.* 32:e23413. 10.1002/ajhb.23413 32222050

[B60] Dours-Zimmermann M. Maurer K. Rauch U. Stoffel W. Fassler R. Zimmermann D. (2009). Versican V2 assembles the extracellular matrix surrounding the nodes of ranvier in the CNS. *J. Neurosci.* 29 7731–7742. 10.1523/JNEUROSCI.4158-08.2009 19535585 PMC6665632

[B61] Eastwood S. Harrison P. (2006). Cellular basis of reduced cortical reelin expression in schizophrenia. *Am. J. Psychiatry* 163 540–542. 10.1176/appi.ajp.163.3.540 16513881

[B62] Enwright J. Sanapala S. Foglio A. Berry R. Fish K. Lewis D. (2016). Reduced labeling of parvalbumin neurons and perineuronal nets in the dorsolateral prefrontal cortex of subjects with schizophrenia. *Neuropsychopharmacology* 41 2206–2214. 10.1038/npp.2016.24 26868058 PMC4946056

[B63] Fagiolini A. Frank E. Houck P. Mallinger A. Swartz H. Buysse D. (2002). Prevalence of obesity and weight change during treatment in patients with bipolar I disorder. *J. Clin. Psychiatry* 63 528–533. 10.4088/jcp.v63n0611 12088166

[B64] Fatemi S. Earle J. McMenomy T. (2000). Reduction in Reelin immunoreactivity in hippocampus of subjects with schizophrenia, bipolar disorder and major depression. *Mol. Psychiatry* 5 654–663. 10.1038/sj.mp.4000783 11126396

[B65] Fawcett J. Kwok J. (2022). Proteoglycan sulphation in the function of the mature central nervous system. *Front. Integr. Neurosci.* 16:895493. 10.3389/fnint.2022.895493 35712345 PMC9195417

[B66] Feldman E. Callaghan B. Pop-Busui R. Zochodne D. Wright D. Bennett D. (2019). Diabetic neuropathy. *Nat. Rev. Dis. Primers* 5:41. 10.1038/s41572-019-0092-1 31197153

[B67] ffrench-Constant C. Colognato H. (2004). Integrins: versatile integrators of extracellular signals. *Trends Cell Biol.* 14 678–686. 10.1016/j.tcb.2004.10.005 15564044

[B68] Frischknecht R. Gundelfinger E. (2012). The brain’s extracellular matrix and its role in synaptic plasticity. *Adv. Exp. Med. Biol.* 970 153–171. 10.1007/978-3-7091-0932-8_7 22351055

[B69] Frischknecht R. Heine M. Perrais D. Seidenbecher C. Choquet D. Gundelfinger E. D. (2009). Brain extracellular matrix affects AMPA receptor lateral mobility and short-term synaptic plasticity. *Nat. Neurosci.* 12 897–904. 10.1038/nn.2338 19483686

[B70] Gach K. Szemraj J. Wyrebska A. Janecka A. (2011). The influence of opioids on matrix metalloproteinase-2 and -9 secretion and mRNA levels in MCF-7 breast cancer cell line. *Mol. Biol. Rep.* 38 1231–1236. 10.1007/s11033-010-0222-z 20563853

[B71] Ganguly K. Rejmak E. Mikosz M. Nikolaev E. Knapska E. Kaczmarek L. (2013). Matrix metalloproteinase (MMP) 9 transcription in mouse brain induced by fear learning. *J. Biol. Chem.* 288 20978–20991. 10.1074/jbc.M113.457903 23720741 PMC3774367

[B72] Garcia-Garcia I. Horstmann A. Jurado M. Garolera M. Chaudhry S. Margulies D. (2014). Reward processing in obesity, substance addiction and non-substance addiction. *Obes. Rev.* 15 853–869. 10.1111/obr.12221 25263466

[B73] Garcia-Keller C. Neuhofer D. Bobadilla A. Spencer S. Chioma V. Monforton C. (2019). Extracellular matrix signaling through beta3 integrin mediates cocaine cue-induced transient synaptic plasticity and relapse. *Biol. Psychiatry.* 86 377–387. 10.1016/j.biopsych.2019.03.982 31126696 PMC6697624

[B74] Garcia-Keller C. Scofield M. Neuhofer D. Varanasi S. Reeves M. Hughes B. (2020). Relapse-associated transient synaptic potentiation requires integrin-mediated activation of focal adhesion kinase and cofilin in D1-expressing neurons. *J. Neurosci.* 40 8463–8477. 10.1523/JNEUROSCI.2666-19.2020 33051346 PMC7605418

[B75] GBD 2015 Obesity Collaborators Afshin A. Forouzanfar M. Reitsma M. Sur P. Estep K. (2017). Health effects of overweight and obesity in 195 countries over 25 years. *N. Engl. J. Med.* 377 13–27. 10.1056/NEJMoa1614362 28604169 PMC5477817

[B76] Giamanco K. Matthews R. (2012). Deconstructing the perineuronal net: cellular contributions and molecular composition of the neuronal extracellular matrix. *Neuroscience* 218 367–384. 10.1016/j.neuroscience.2012.05.055 22659016 PMC3400135

[B77] Gisabella B. Babu J. Valeri J. Rexrode L. Pantazopoulos H. (2021). Sleep and memory consolidation dysfunction in psychiatric disorders: evidence for the involvement of extracellular matrix molecules. *Front. Neurosci.* 15:646678. 10.3389/fnins.2021.646678 34054408 PMC8160443

[B78] Gogolla N. Caroni P. Luthi A. Herry C. (2009). Perineuronal nets protect fear memories from erasure. *Science* 325 1258–1261. 10.1126/science.1174146 19729657

[B79] Goldstein R. Volkow N. (2011). Dysfunction of the prefrontal cortex in addiction: neuroimaging findings and clinical implications. *Nat. Rev. Neurosci.* 12 652–669. 10.1038/nrn3119 22011681 PMC3462342

[B80] Grant B. Saha T. Ruan W. Goldstein R. Chou S. Jung J. (2016). Epidemiology of DSM-5 drug use disorder: results from the national epidemiologic survey on alcohol and related conditions-III. *JAMA Psychiatry* 73 39–47. 10.1001/jamapsychiatry.2015.2132 26580136 PMC5062605

[B81] Guarque-Chabrera J. Sanchez-Hernandez A. Ibanez-Marin P. Melchor-Eixea I. Miquel M. (2022). Role of perineuronal nets in the cerebellar cortex in cocaine-induced conditioned preference, extinction, and reinstatement. *Neuropharmacology* 218:109210. 10.1016/j.neuropharm.2022.109210 35985392

[B82] Guillemot-Legris O. Muccioli G. (2017). Obesity-induced neuroinflammation: beyond the hypothalamus. *Trends Neurosci.* 40 237–253. 10.1016/j.tins.2017.02.005 28318543

[B83] Gundelfinger E. Frischknecht R. Choquet D. Heine M. (2010). Converting juvenile into adult plasticity: a role for the brain’s extracellular matrix. *Eur. J. Neurosci.* 31 2156–2165. 10.1111/j.1460-9568.2010.07253.x 20497467

[B84] Gustafson D. Karlsson C. Skoog I. Rosengren L. Lissner L. Blennow K. (2007). Mid-life adiposity factors relate to blood-brain barrier integrity in late life. *J. Intern. Med.* 262 643–650. 10.1111/j.1365-2796.2007.01869.x 17986201

[B85] Hao S. Dey A. Yu X. Stranahan A. (2016). Dietary obesity reversibly induces synaptic stripping by microglia and impairs hippocampal plasticity. *Brain Behav. Immun.* 51 230–239. 10.1016/j.bbi.2015.08.023 26336035 PMC4679537

[B86] Harkness J. Bushana P. Todd R. Clegern W. Sorg B. Wisor J. (2019). Sleep disruption elevates oxidative stress in parvalbumin-positive cells of the rat cerebral cortex. *Sleep* 42:zsy201. 10.1093/sleep/zsy201 30371896 PMC6335871

[B87] Hartig W. Brauer K. Bigl V. Bruckner G. (1994). Chondroitin sulfate proteoglycan-immunoreactivity of lectin-labeled perineuronal nets around parvalbumin-containing neurons. *Brain Res.* 635 307–311. 10.1016/0006-8993(94)91452-4 8173967

[B88] Hartig W. Derouiche A. Welt K. Brauer K. Grosche J. Mader M. (1999). Cortical neurons immunoreactive for the potassium channel Kv3.1b subunit are predominantly surrounded by perineuronal nets presumed as a buffering system for cations. *Brain Res.* 842 15–29. 10.1016/s0006-8993(99)01784-9 10526091

[B89] Hartig W. Meinicke A. Michalski D. Schob S. Jager C. (2022). Update on perineuronal net staining with wisteria floribunda agglutinin (WFA). *Front. Integr. Neurosci.* 16:851988. 10.3389/fnint.2022.851988 35431825 PMC9011100

[B90] Hinder L. Murdock B. Park M. Bender D. O’Brien P. Rumora A. (2018). Transcriptional networks of progressive diabetic peripheral neuropathy in the db/db mouse model of type 2 diabetes: an inflammatory story. *Exp. Neurol.* 305 33–43. 10.1016/j.expneurol.2018.03.011 29550371 PMC5955815

[B91] Hu X. Yang W. Zhang M. Du L. Tian J. Zhu J. (2021). Glial IL-33 signaling through an ST2-to-CXCL12 pathway in the spinal cord contributes to morphine-induced hyperalgesia and tolerance. *Sci. Signal.* 14:eabe3773. 10.1126/scisignal.abe3773 34516755

[B92] Hume C. Massey S. van den Buuse M. (2020). The effect of chronic methamphetamine treatment on schizophrenia endophenotypes in heterozygous reelin mice: implications for schizophrenia. *Biomolecules* 10:940. 10.3390/biom10060940 32580454 PMC7355789

[B93] Ishiguro H. Hall F. Horiuchi Y. Sakurai T. Hishimoto A. Grumet M. (2014). NrCAM-regulating neural systems and addiction-related behaviors. *Addict. Biol.* 19 343–353. 10.1111/j.1369-1600.2012.00469.x 22780223 PMC3470748

[B94] Ishii M. Maeda N. (2008). Oversulfated chondroitin sulfate plays critical roles in the neuronal migration in the cerebral cortex. *J. Biol. Chem.* 283 32610–32620. 10.1074/jbc.M806331200 18819920

[B95] Janak P. Chaudhri N. (2010). The potent effect of environmental context on relapse to alcohol-seeking after extinction. *Open Addict. J.* 3 76–87. 10.2174/1874941001003010076 21132088 PMC2995923

[B96] Jensen T. Karlsson P. Gylfadottir S. Andersen S. Bennett D. Tankisi H. (2021). Painful and non-painful diabetic neuropathy, diagnostic challenges and implications for future management. *Brain.* 144 1632–1645. 10.1093/brain/awab079 33711103 PMC8320269

[B97] Jimenez D. Thomas L. Bartels S. (2019). The role of serious mental illness in motivation, participation and adoption of health behavior change among obese/sedentary Latino adults. *Ethn. Health* 24 889–896. 10.1080/13557858.2017.1390552 29124951 PMC6224308

[B98] Jones C. (2013). Heroin use and heroin use risk behaviors among nonmedical users of prescription opioid pain relievers - United States, 2002-2004 and 2008-2010. *Drug Alcohol Depend.* 132 95–100. 10.1016/j.drugalcdep.2013.01.007 23410617

[B99] Kalb R. Hockfield S. (1988). Molecular evidence for early activity-dependent development of hamster motor neurons. *J. Neurosci.* 8 2350–2360. 10.1523/JNEUROSCI.08-07-02350.1988 3249230 PMC6569534

[B100] Karus M. Samtleben S. Busse C. Tsai T. Dietzel I. Faissner A. (2012). Normal sulfation levels regulate spinal cord neural precursor cell proliferation and differentiation. *Neural Dev.* 7:20. 10.1186/1749-8104-7-20 22681904 PMC3423038

[B101] Kelley K. Dantzer R. (2011). Alcoholism and inflammation: neuroimmunology of behavioral and mood disorders. *Brain Behav. Immun.* 25 Suppl. 1 S13–S20. 10.1016/j.bbi.2010.12.013 21193024 PMC4068736

[B102] Kohnke S. Buller S. Nuzzaci D. Ridley K. Lam B. Pivonkova H. (2021). Nutritional regulation of oligodendrocyte differentiation regulates perineuronal net remodeling in the median eminence. *Cell Rep.* 36:109362. 10.1016/j.celrep.2021.109362 34260928 PMC8293628

[B103] Kohno M. Link J. Dennis L. McCready H. Huckans M. Hoffman W. (2019). Neuroinflammation in addiction: a review of neuroimaging studies and potential immunotherapies. *Pharmacol. Biochem. Behav.* 179 34–42. 10.1016/j.pbb.2019.01.007 30695700 PMC6637953

[B104] Kruyer A. Chioma V. Kalivas P. (2020). The opioid-addicted tetrapartite synapse. *Biol. Psychiatry* 87 34–43. 10.1016/j.biopsych.2019.05.025 31378302 PMC6898767

[B105] Kutlu M. Gould T. (2016). Effects of drugs of abuse on hippocampal plasticity and hippocampus-dependent learning and memory: contributions to development and maintenance of addiction. *Learn. Mem.* 23 515–533. 10.1101/lm.042192.116 27634143 PMC5026208

[B106] Lasek A. (2016). Effects of ethanol on brain extracellular matrix: implications for alcohol use disorder. *Alcohol Clin. Exp. Res.* 40 2030–2042. 10.1111/acer.13200 27581478 PMC5048555

[B107] Lendvai D. Morawski M. Bruckner G. Negyessy L. Baksa G. Glasz T. (2012). Perisynaptic aggrecan-based extracellular matrix coats in the human lateral geniculate body devoid of perineuronal nets. *J. Neurosci. Res.* 90 376–387. 10.1002/jnr.22761 21959900

[B108] Lewandowski S. Hodebourg R. Wood S. Carter J. Nelson K. Kalivas P. (2023). Matrix metalloproteinase activity during methamphetamine cued relapse. *Addict. Biol.* 28:e13279. 10.1111/adb.13279 37186441 PMC10506177

[B109] Lin R. Rosahl T. Whiting P. Fawcett J. Kwok J. (2011). 6-Sulphated chondroitins have a positive influence on axonal regeneration. *PLoS One* 6:e21499. 10.1371/journal.pone.0021499 21747937 PMC3128591

[B110] Liu W. Han Y. Liu Y. Song A. Barnes B. Song X. (2010). Spinal matrix metalloproteinase-9 contributes to physical dependence on morphine in mice. *J. Neurosci.* 30 7613–7623. 10.1523/JNEUROSCI.1358-10.2010 20519536 PMC3842477

[B111] Lowe P. Morel C. Ambade A. Iracheta-Vellve A. Kwiatkowski E. Satishchandran A. (2020). Chronic alcohol-induced neuroinflammation involves CCR2/5-dependent peripheral macrophage infiltration and microglia alterations. *J. Neuroinflamm.* 17:296. 10.1186/s12974-020-01972-5 33036616 PMC7547498

[B112] Lu L. Grimm J. Hope B. Shaham Y. (2004). Incubation of cocaine craving after withdrawal: a review of preclinical data. *Neuropharmacology* 47 Suppl. 1 214–226. 10.1016/j.neuropharm.2004.06.027 15464139

[B113] Lupori L. Totaro V. Cornuti S. Ciampi L. Carrara F. Grilli E. (2023). A comprehensive atlas of perineuronal net distribution and colocalization with parvalbumin in the adult mouse brain. *bioRxiv [Preprint]* 10.1101/2023.01.24.525313 37436896

[B114] Luppino F. de Wit L. Bouvy P. Stijnen T. Cuijpers P. Penninx B. (2010). Overweight, obesity, and depression: a systematic review and meta-analysis of longitudinal studies. *Arch. Gen. Psychiatry* 67 220–229. 10.1001/archgenpsychiatry.2010.2 20194822

[B115] Ly C. Greb A. Cameron L. Wong J. Barragan E. Wilson P. (2018). Psychedelics promote structural and functional neural plasticity. *Cell Rep.* 23 3170–3182. 10.1016/j.celrep.2018.05.022 29898390 PMC6082376

[B116] Maeda N. (2010). Structural variation of chondroitin sulfate and its roles in the central nervous system. *Cent. Nerv. Syst. Agents Med. Chem.* 10 22–31. 20236040 10.2174/187152410790780136

[B117] Maeda N. (2015). Proteoglycans and neuronal migration in the cerebral cortex during development and disease. *Frontiers in neuroscience.* 9:98. 10.3389/fnins.2015.00098 25852466 PMC4369650

[B118] Maeda N. Ishii M. Nishimura K. Kamimura K. (2011). Functions of chondroitin sulfate and heparan sulfate in the developing brain. *Neurochem. Res.* 36 1228–1240. 10.1007/s11064-010-0324-y 21110089

[B119] Mafi A. Hofer L. Russ M. Young J. Mellott J. (2020). The density of perineuronal nets increases with age in the inferior colliculus in the fischer brown norway rat. *Front. Aging Neurosci.* 12:27. 10.3389/fnagi.2020.00027 32116654 PMC7026493

[B120] Main M. Rao S. O’Keefe J. (2010). Trends in obesity and extreme obesity among US adults. *JAMA* 303 1695;authorrely–6. 10.1001/jama.2010.517 20442381

[B121] Maiya R. Zhou Y. Norris E. Kreek M. Strickland S. (2009). Tissue plasminogen activator modulates the cellular and behavioral response to cocaine. *Proc. Natl. Acad. Sci. U.S.A.* 106 1983–1988. 10.1073/pnas.0812491106 19181855 PMC2644150

[B122] Marchant N. (2019). Break the net, break the cycle: removal of perineuronal nets in the lateral hypothalamus decreases cocaine relapse. *Neuropsychopharmacology* 44 835–836. 10.1038/s41386-018-0245-z 30867569 PMC6461892

[B123] Martin A. Davidson T. (2014). Human cognitive function and the obesogenic environment. *Physiol. Behav.* 136 185–193. 10.1016/j.physbeh.2014.02.062 24631299 PMC4161661

[B124] Martin D. Nichols C. (2016). Psychedelics recruit multiple cellular types and produce complex transcriptional responses within the brain. *Ebiomedicine.* 11 262–277. 10.1016/j.ebiom.2016.08.049 27649637 PMC5050000

[B125] Mascio G. Notartomaso S. Martinello K. Liberatore F. Bucci D. Imbriglio T. (2022). A progressive build-up of perineuronal nets in the somatosensory cortex is associated with the development of chronic pain in mice. *J. Neurosci.* 42 3037–3048. 10.1523/JNEUROSCI.1714-21.2022 35193928 PMC8985861

[B126] Mash D. ffrench-Mullen J. Adi N. Qin Y. Buck A. Pablo J. (2007). Gene expression in human hippocampus from cocaine abusers identifies genes which regulate extracellular matrix remodeling. *PLoS One* 2:e1187. 10.1371/journal.pone.0001187 18000554 PMC2063513

[B127] Matikainen-Ankney B. Kravitz A. (2018). Persistent effects of obesity: a neuroplasticity hypothesis. *Ann. N.Y. Acad. Sci.* 1428 221–239. 10.1111/nyas.13665 29741270 PMC6158064

[B128] Mauney S. Athanas K. Pantazopoulos H. Shaskan N. Passeri E. Berretta S. (2013). Developmental pattern of perineuronal nets in the human prefrontal cortex and their deficit in schizophrenia. *Biol. Psychiatry* 74 427–435. 10.1016/j.biopsych.2013.05.007 23790226 PMC3752333

[B129] McClernon F. Conklin C. Kozink R. Adcock R. Sweitzer M. Addicott M. (2016). Hippocampal and insular response to smoking-related environments: neuroimaging evidence for drug-context effects in nicotine dependence. *Neuropsychopharmacology* 41 877–885. 10.1038/npp.2015.214 26179147 PMC4707833

[B130] Mezu-Ndubuisi O. Maheshwari A. (2021). The role of integrins in inflammation and angiogenesis. *Pediatr. Res.* 89 1619–1626. 10.1038/s41390-020-01177-9 33027803 PMC8249239

[B131] Miguel-Hidalgo J. (2023). Astrocytes as context for the involvement of myelin and nodes of ranvier in the pathophysiology of depression and stress-related disorders. *J. Psychiatr. Brain Sci.* 8:e230001. 10.20900/jpbs.20230001 36866235 PMC9976698

[B132] Milev P. Maurel P. Chiba A. Mevissen M. Popp S. Yamaguchi Y. (1998). Differential regulation of expression of hyaluronan-binding proteoglycans in developing brain: aggrecan, versican, neurocan, and brevican. *Biochem. Biophys. Res. Commun.* 247 207–212. 10.1006/bbrc.1998.8759 9642104

[B133] Miller A. Spencer S. (2014). Obesity and neuroinflammation: a pathway to cognitive impairment. *Brain Behav. Immun.* 42 10–21. 10.1016/j.bbi.2014.04.001 24727365

[B134] Mirzadeh Z. Alonge K. Cabrales E. Herranz-Perez V. Scarlett J. Brown J. (2019). Perineuronal net formation during the critical period for neuronal maturation in the hypothalamic arcuate nucleus. *Nat. Metab.* 1 212–221. 10.1038/s42255-018-0029-0 31245789 PMC6594569

[B135] Miyata S. Komatsu Y. Yoshimura Y. Taya C. Kitagawa H. (2012). Persistent cortical plasticity by upregulation of chondroitin 6-sulfation. *Nat. Neurosci.* 15 S1–S2. 10.1038/nn.3023 22246436

[B136] Mizoguchi H. Yamada K. Mouri A. Niwa M. Mizuno T. Noda Y. (2007). Role of matrix metalloproteinase and tissue inhibitor of MMP in methamphetamine-induced behavioral sensitization and reward: implications for dopamine receptor down-regulation and dopamine release. *J. Neurochem.* 102 1548–1560. 10.1111/j.1471-4159.2007.04623.x 17472698

[B137] Mohamedi Y. Fontanil T. Cobo T. Cal S. Obaya A. (2020). New insights into ADAMTS metalloproteases in the central nervous system. *Biomolecules* 10:403. 10.3390/biom10030403 32150898 PMC7175268

[B138] Morawski M. Bruckner G. Jager C. Seeger G. Arendt T. (2010). Neurons associated with aggrecan-based perineuronal nets are protected against tau pathology in subcortical regions in Alzheimer’s disease. *Neuroscience* 169 1347–1363. 10.1016/j.neuroscience.2010.05.022 20497908

[B139] Morawski M. Bruckner M. Riederer P. Bruckner G. Arendt T. (2004). Perineuronal nets potentially protect against oxidative stress. *Exp. Neurol.* 188 309–315. 10.1016/j.expneurol.2004.04.017 15246831

[B140] Moulton E. Elman I. Becerra L. Goldstein R. Borsook D. (2014). The cerebellum and addiction: insights gained from neuroimaging research. *Addict. Biol.* 19 317–331. 10.1111/adb.12101 24851284 PMC4031616

[B141] Nadanaka S. Miyata S. Yaqiang B. Tamura J. Habuchi O. Kitagawa H. (2020). Reconsideration of the Semaphorin-3A binding motif found in chondroitin sulfate using Galnac4s-6st-Knockout mice. *Biomolecules* 10:1499. 10.3390/biom10111499 33143303 PMC7694144

[B142] Nagy V. Bozdagi O. Huntley G. (2007). The extracellular protease matrix metalloproteinase-9 is activated by inhibitory avoidance learning and required for long-term memory. *Learn. Mem.* 14 655–664. 10.1101/lm.678307 17909100 PMC2044557

[B143] Nakamoto K. Kawasaki S. Kobori T. Fujita-Hamabe W. Mizoguchi H. Yamada K. (2012). Involvement of matrix metalloproteinase-9 in the development of morphine tolerance. *Eur. J. Pharmacol.* 683 86–92. 10.1016/j.ejphar.2012.03.006 22445883

[B144] Narendran R. Lopresti B. Mason N. Deuitch L. Paris J. Himes M. (2014). Cocaine abuse in humans is not associated with increased microglial activation: an 18-kDa translocator protein positron emission tomography imaging study with [11C]PBR28. *J. Neurosci.* 34 9945–9950. 10.1523/jneurosci.0928-14.2014 25057196 PMC4107410

[B145] Natarajan R. Harding J. Wright J. W. (2013). A role for matrix metalloproteinases in nicotine-induced conditioned place preference and relapse in adolescent female rats. *J. Exp. Neurosci.* 7 1–14. 10.4137/JEN.S11381 25157203 PMC4089657

[B146] Negus S. Freeman K. (2018). Abuse potential of biased mu opioid receptor agonists. *Trends Pharmacol. Sci.* 39 916–919. 10.1016/j.tips.2018.08.007 30343727 PMC8174448

[B147] Nestler E. (2005). The neurobiology of cocaine addiction. *Sci. Pract. Perspect.* 3 4–10. 10.1151/spp05314 18552739 PMC2851032

[B148] Nguyen P. Dorman L. Pan S. Vainchtein I. Han R. Nakao-Inoue H. (2020). Microglial remodeling of the extracellular matrix promotes synapse plasticity. *Cell* 182 388–403.e15. 10.1016/j.cell.2020.05.050 32615087 PMC7497728

[B149] Niaura R. Rohsenow D. Binkoff J. Monti P. Pedraza M. Abrams D. (1988). Relevance of cue reactivity to understanding alcohol and smoking relapse. *J. Abnorm. Psychol.* 97 133–152. 10.1037//0021-843x.97.2.133 3290304

[B150] Nichols D. (2016). Psychedelics. *Pharmacol. Rev.* 68 264–355. 10.1124/pr.115.011478 26841800 PMC4813425

[B151] Noh H. Lee H. Kim D. Kang S. Cho G. Rho J. (2004). A cDNA microarray analysis of gene expression profiles in rat hippocampus following a ketogenic diet. *Brain Res. Mol. Brain Res.* 129 80–87. 10.1016/j.molbrainres.2004.06.020 15469884

[B152] Norwitz N. Sethi S. Palmer C. (2020). Ketogenic diet as a metabolic treatment for mental illness. *Curr. Opin. Endocrinol. Diabetes Obes.* 27 269–274. 10.1097/MED.0000000000000564 32773571

[B153] O’Connor A. Burton T. Mansuri H. Hand G. Leamey C. Sawatari A. (2019). Environmental enrichment from birth impacts parvalbumin expressing cells and wisteria floribunda agglutinin labelled peri-neuronal nets within the developing murine striatum. *Front. Neuroanat.* 13:90. 10.3389/fnana.2019.00090 31708753 PMC6821641

[B154] Oliva I. Wanat M. (2016). Ventral tegmental area afferents and drug-dependent behaviors. *Front. Psychiatry* 7:30. 10.3389/fpsyt.2016.00030 27014097 PMC4780106

[B155] Oltman C. Coppey L. Gellett J. Davidson E. Lund D. Yorek M. (2005). Progression of vascular and neural dysfunction in sciatic nerves of Zucker diabetic fatty and Zucker rats. *Am. J. Physiol. Endocrinol. Metab.* 289 E113–E122. 10.1152/ajpendo.00594.2004 15727946

[B156] Oommen A. Roberts K. Joshi L. Cunningham S. (2023). Transcriptomic analysis of glycosylation and neuroregulatory pathways in rodent models in response to psychedelic molecules. *Int. J. Mol. Sci.* 24:1200. 10.3390/ijms24021200 36674723 PMC9867456

[B157] Page-McCaw A. Ewald A. Werb Z. (2007). Matrix metalloproteinases and the regulation of tissue remodelling. *Nat. Rev. Mol. Cell Biol.* 8 221–233. 10.1038/nrm2125 17318226 PMC2760082

[B158] Pantazopoulos H. Gisabella B. Rexrode L. Benefield D. Yildiz E. Seltzer P. (2020). Circadian rhythms of perineuronal net composition. *eNeuro* 7 ENEURO.34–ENEURO.19. 10.1523/ENEURO.0034-19.2020 32719104 PMC7405073

[B159] Pantazopoulos H. Hossain N. Chelini G. Durning P. Barbas H. Zikopoulos B. (2022). Chondroitin sulphate proteoglycan axonal coats in the human mediodorsal thalamic nucleus. *Front. Integr. Neurosci.* 16:934764. 10.3389/fnint.2022.934764 35875507 PMC9298528

[B160] Pantazopoulos H. Lange N. Hassinger L. Berretta S. (2006). Subpopulations of neurons expressing parvalbumin in the human amygdala. *J. Comp. Neurol.* 496 706–722. 10.1002/cne.20961 16615121 PMC1927834

[B161] Pantazopoulos H. Markota M. Jaquet F. Ghosh D. Wallin A. Santos A. (2015). Aggrecan and chondroitin-6-sulfate abnormalities in schizophrenia and bipolar disorder: a postmortem study on the amygdala. *Transl. Psychiatry* 5:e496. 10.1038/tp.2014.128 25603412 PMC4312825

[B162] Pantazopoulos H. Woo T. Lim M. Lange N. Berretta S. (2010). Extracellular matrix-glial abnormalities in the amygdala and entorhinal cortex of subjects diagnosed with schizophrenia. *Arch. Gen. Psychiatry* 67 155–166. 10.1001/archgenpsychiatry.2009.196 20124115 PMC4208310

[B163] Parent M. Darling J. Henderson Y. (2014). Remembering to eat: hippocampal regulation of meal onset. *Am. J. Physiol. Regul. Integr. Comp. Physiol.* 306 R701–R713. 10.1152/ajpregu.00496.2013 24573183 PMC4025066

[B164] Peters A. Sherman L. (2020). Diverse roles for hyaluronan and hyaluronan receptors in the developing and adult nervous system. *Int. J. Mol. Sci.* 21 5988. 10.3390/ijms21175988 32825309 PMC7504301

[B165] Phillips J. Norris S. Talbot J. Birmingham M. Hatchard T. Ortiz A. (2019). Single, repeated, and maintenance ketamine infusions for treatment-resistant depression: a randomized controlled trial. *Am. J. Psychiatry* 176 401–409. 10.1176/appi.ajp.2018.18070834 30922101

[B166] Pizzorusso T. Medini P. Berardi N. Chierzi S. Fawcett J. Maffei L. (2002). Reactivation of ocular dominance plasticity in the adult visual cortex. *Science* 298 1248–1251. 10.1126/science.1072699 12424383

[B167] Powers A. III Gancsos M. Finn E. Morgan P. Corlett P. (2015). Ketamine-induced hallucinations. *Psychopathology* 48 376–385. 10.1159/000438675 26361209 PMC4684980

[B168] Quintero G. (2013). Role of nucleus accumbens glutamatergic plasticity in drug addiction. *Neuropsychiatr. Dis. Treat.* 9 1499–1512. 10.2147/NDT.S45963 24109187 PMC3792955

[B169] Ray M. Williams B. Kuppe M. Bryant C. Logan R. W. A. (2022). Glitch in the matrix: the role of extracellular matrix remodeling in opioid use disorder. *Front. Integr. Neurosci.* 16:899637. 10.3389/fnint.2022.899637 35757099 PMC9218427

[B170] Reichelt A. Lemieux C. Princz-Lebel O. Singh A. Bussey T. Saksida L. (2021). Age-dependent and region-specific alteration of parvalbumin neurons, perineuronal nets and microglia in the mouse prefrontal cortex and hippocampus following obesogenic diet consumption. *Sci. Rep.* 11:5593. 10.1038/s41598-021-85092-x 33692414 PMC7970944

[B171] Rieser N. Herdener M. Preller K. (2022). Psychedelic-assisted therapy for substance use disorders and potential mechanisms of action. *Curr. Top. Behav. Neurosci.* 56 187–211. 10.1007/7854_2021_284 34910289

[B172] Riga D. Kramvis I. Koskinen M. van Bokhoven P. van der Harst J. Heistek T. (2017). Hippocampal extracellular matrix alterations contribute to cognitive impairment associated with a chronic depressive-like state in rats. *Sci. Transl. Med.* 9:eaai8753. 10.1126/scitranslmed.aai8753 29263233

[B173] Rivera S. Khrestchatisky M. Kaczmarek L. Rosenberg G. Jaworski D. (2010). Metzincin proteases and their inhibitors: foes or friends in nervous system physiology? *J. Neurosci.* 30 15337–15357. 10.1523/JNEUROSCI.3467-10.2010 21084591 PMC3072038

[B174] Rogers S. Rankin-Gee E. Risbud R. Porter B. Marsh E. (2018). Normal development of the perineuronal net in humans; in patients with and without epilepsy. *Neuroscience* 384 350–360. 10.1016/j.neuroscience.2018.05.039 29885523 PMC6062204

[B175] Rossier J. Bernard A. Cabungcal J. Perrenoud Q. Savoye A. Gallopin T. (2015). Cortical fast-spiking parvalbumin interneurons enwrapped in the perineuronal net express the metallopeptidases Adamts8, Adamts15 and Neprilysin. *Mol. Psychiatry* 20 154–161. 10.1038/mp.2014.162 25510509 PMC4356748

[B176] Roura-Martinez D. Diaz-Bejarano P. Ucha M. Paiva R. Ambrosio E. Higuera-Matas A. (2020). Comparative analysis of the modulation of perineuronal nets in the prefrontal cortex of rats during protracted withdrawal from cocaine, heroin and sucrose self-administration. *Neuropharmacology* 180:108290. 10.1016/j.neuropharm.2020.108290 32888961

[B177] Rucker J. Jelen L. Flynn S. Frowde K. Young A. (2016). Psychedelics in the treatment of unipolar mood disorders: a systematic review. *J. Psychopharmacol.* 30 1220–1229. 10.1177/0269881116679368 27856684

[B178] Saksela O. (1985). Plasminogen activation and regulation of pericellular proteolysis. *Biochim. Biophys. Acta* 823 35–65. 10.1016/0304-419x(85)90014-9 2413894

[B179] SAMHSA (2018). *Key substance use and mental health indicators in the United States: results from the 2017 national survey on drug use and health.* Rockville, MD: SAMHSA.

[B180] Samochowiec A. Grzywacz A. Kaczmarek L. Bienkowski P. Samochowiec J. Mierzejewski P. (2010). Functional polymorphism of matrix metalloproteinase-9 (MMP-9) gene in alcohol dependence: family and case control study. *Brain Res.* 1327 103–106. 10.1016/j.brainres.2010.02.072 20197064

[B181] Samuel V. Rajeev T. Ramesh L. Sundararaman A. (2023). Integrin receptor trafficking in health and disease. *Prog. Mol. Biol. Transl. Sci.* 196 271–302. 10.1016/bs.pmbts.2022.09.008 36813362

[B182] Sarnyai Z. Palmer C. (2020). Ketogenic therapy in serious mental illness: emerging evidence. *Int. J. Neuropsychopharmacol.* 23 434–439. 10.1093/ijnp/pyaa036 32573722 PMC7387764

[B183] Scarlett J. Hu S. Alonge K. (2022). The “Loss” of perineuronal nets in Alzheimer’s disease: missing or hiding in plain sight? *Front. Integr. Neurosci.* 16:896400. 10.3389/fnint.2022.896400 35694184 PMC9174696

[B184] Scarlett J. Rojas J. Matsen M. Kaiyala K. Stefanovski D. Bergman R. (2016). Central injection of fibroblast growth factor 1 induces sustained remission of diabetic hyperglycemia in rodents. *Nat. Med.* 22 800–806. 10.1038/nm.4101 27213816 PMC4938755

[B185] Schuster T. Krug M. Stalder M. Hackel N. Gerardy-Schahn R. Schachner M. (2001). Immunoelectron microscopic localization of the neural recognition molecules L1, NCAM, and its isoform NCAM180, the NCAM-associated polysialic acid, beta1 integrin and the extracellular matrix molecule tenascin-R in synapses of the adult rat hippocampus. *J. Neurobiol.* 49 142–158. 10.1002/neu.1071 11598921

[B186] Schwartz N. Domowicz M. (2023). Chemistry and function of glycosaminoglycans in the nervous system. *Adv. Neurobiol.* 29 117–162. 10.1007/978-3-031-12390-0_5 36255674

[B187] Seney M. Kim S. Glausier J. Hildebrand M. Xue X. Zong W. (2021). Transcriptional alterations in dorsolateral prefrontal cortex and nucleus accumbens implicate neuroinflammation and synaptic remodeling in opioid use disorder. *Biol. Psychiatry* 90 550–562. 10.1016/j.biopsych.2021.06.007 34380600 PMC8463497

[B188] Shao L. Liao C. Gregg I. Davoudian P. Savalia N. Delagarza K. (2021). Psilocybin induces rapid and persistent growth of dendritic spines in frontal cortex in vivo. *Neuron* 109 2535–44.e4. 10.1016/j.neuron.2021.06.008 34228959 PMC8376772

[B189] Shin C. Kim Y. (2020). Ketamine in major depressive disorder: mechanisms and future perspectives. *Psychiatry Investig.* 17 181–192. 10.30773/pi.2019.0236 32209965 PMC7113176

[B190] Siddiqui N. Oshima K. Hippensteel J. (2022). Proteoglycans and glycosaminoglycans in central nervous system injury. *Am. J. Physiol. Cell Physiol.* 323 C46–C55. 10.1152/ajpcell.00053.2022 35613357 PMC9273265

[B191] Silver D. Silver J. (2014). Contributions of chondroitin sulfate proteoglycans to neurodevelopment, injury, and cancer. *Curr. Opin. Neurobiol.* 27 171–178. 10.1016/j.conb.2014.03.016 24762654 PMC4122631

[B192] Slaker M. Barnes J. Sorg B. Grimm J. (2016a). Impact of environmental enrichment on perineuronal nets in the prefrontal cortex following early and late abstinence from sucrose self-administration in rats. *PLoS One* 11:e0168256. 10.1371/journal.pone.0168256 27977779 PMC5158028

[B193] Slaker M. Blacktop J. Sorg B. (2016b). Caught in the net: perineuronal nets and addiction. *Neural Plast.* 2016:7538208. 10.1155/2016/7538208 26904301 PMC4745418

[B194] Slaker M. Churchill L. Todd R. Blacktop J. Zuloaga D. Raber J. (2015). Removal of perineuronal nets in the medial prefrontal cortex impairs the acquisition and reconsolidation of a cocaine-induced conditioned place preference memory. *J. Neurosci.* 35 4190–4202. 10.1523/JNEUROSCI.3592-14.2015 25762666 PMC4355195

[B195] Slaker M. Jorgensen E. Hegarty D. Liu X. Kong Y. Zhang F. (2018). Cocaine exposure modulates perineuronal nets and synaptic excitability of fast-spiking interneurons in the medial prefrontal cortex. *eNeuro* 5:ENEURO.221–ENEURO.218. 10.1523/ENEURO.0221-18.2018 30294670 PMC6171740

[B196] Sloshower J. Skosnik P. Safi-Aghdam H. Pathania S. Syed S. Pittman B. (2023). Psilocybin-assisted therapy for major depressive disorder: an exploratory placebo-controlled, fixed-order trial. *J. Psychopharmacol.* 2698811231154852. 10.1177/02698811231154852 [Epub ahead of print]. 36938991

[B197] Smith A. Kupchik Y. Scofield M. Gipson C. Wiggins A. Thomas C. (2014). Synaptic plasticity mediating cocaine relapse requires matrix metalloproteinases. *Nat. Neurosci.* 17 1655–1657. 10.1038/nn.3846 25326689 PMC4241163

[B198] Smith A. Nealey K. Wright J. Walker B. (2011). Plasticity associated with escalated operant ethanol self-administration during acute withdrawal in ethanol-dependent rats requires intact matrix metalloproteinase systems. *Neurobiol. Learn. Mem.* 96 199–206. 10.1016/j.nlm.2011.04.011 21530666 PMC3148339

[B199] Smith A. Scofield M. Kalivas P. (2015). The tetrapartite synapse: extracellular matrix remodeling contributes to corticoaccumbens plasticity underlying drug addiction. *Brain Res.* 1628(Pt. A) 29–39. 10.1016/j.brainres.2015.03.027 25838241 PMC4589426

[B200] Smith-Thomas L. Stevens J. Fok-Seang J. Faissner A. Rogers J. Fawcett J. (1995). Increased axon regeneration in astrocytes grown in the presence of proteoglycan synthesis inhibitors. *J. Cell Sci.* 108(Pt. 3) 1307–1315. 10.1242/jcs.108.3.1307 7622613

[B201] Spencer M. Minino A. Warner M. (2022). Drug overdose deaths in the United States, 2001-2021. *NCHS Data Brief* 457, 1–8. 36598401

[B202] Steullet P. Cabungcal J. Bukhari S. Ardelt M. Pantazopoulos H. Hamati F. (2018). The thalamic reticular nucleus in schizophrenia and bipolar disorder: role of parvalbumin-expressing neuron networks and oxidative stress. *Mol. Psychiatry.* 23 2057–2065. 10.1038/mp.2017.230 29180672 PMC5972042

[B203] Strous J. Weeland C. van der Draai F. Daams J. Denys D. Lok A. (2022). Brain changes associated with long-term ketamine abuse, a systematic review. *Front. Neuroanat.* 16:795231. 10.3389/fnana.2022.795231 35370568 PMC8972190

[B204] Sugiyama S. Di Nardo A. Aizawa S. Matsuo I. Volovitch M. Prochiantz A. (2008). Experience-dependent transfer of Otx2 homeoprotein into the visual cortex activates postnatal plasticity. *Cell* 134 508–520. 10.1016/j.cell.2008.05.054 18692473

[B205] Susuki K. Chang K. Zollinger D. Liu Y. Ogawa Y. Eshed-Eisenbach Y. (2013). Three mechanisms assemble central nervous system nodes of Ranvier. *Neuron* 78 469–482. 10.1016/j.neuron.2013.03.005 23664614 PMC3756512

[B206] Tabet A. Apra C. Stranahan A. Anikeeva P. (2022). Changes in brain neuroimmunology following injury and disease. *Front. Integr. Neurosci.* 16:894500. 10.3389/fnint.2022.894500 35573444 PMC9093707

[B207] Tajerian M. Clark J. (2015). The role of the extracellular matrix in chronic pain following injury. *Pain* 156 366–370. 10.1097/01.j.pain.0000460323.80020.9d 25679468

[B208] Talin P. Sanabria E. (2017). Ayahuasca’s entwined efficacy: an ethnographic study of ritual healing from ‘addiction’. *Int. J. Drug Policy* 44 23–30. 10.1016/j.drugpo.2017.02.017 28432902 PMC5773453

[B209] Tansley S. Gu N. Guzman A. Cai W. Wong C. Lister K. (2022). Microglia-mediated degradation of perineuronal nets promotes pain. *Science* 377 80–86. 10.1126/science.abl6773 35617374

[B210] Thomsen M. Routhe L. Moos T. (2017). The vascular basement membrane in the healthy and pathological brain. *J. Cereb. Blood Flow Metab.* 37 3300–3317. 10.1177/0271678X17722436 28753105 PMC5624399

[B211] Tran A. Silver J. (2021). Cathepsins in neuronal plasticity. *Neural Regen. Res.* 16 26–35. 10.4103/1673-5374.286948 32788444 PMC7818855

[B212] Trevino-Alvarez A. Sanchez-Ruiz J. Barrera F. Rodriguez-Bautista M. Romo-Nava F. McElroy S. (2023). Weight changes in adults with major depressive disorder: a systematic review and meta-analysis of prospective studies. *J. Affect. Disord.* 332 1–8. 10.1016/j.jad.2023.03.050 36963517

[B213] Tsuji K. Aoki T. Tejima E. Arai K. Lee S. Atochin D. (2005). Tissue plasminogen activator promotes matrix metalloproteinase-9 upregulation after focal cerebral ischemia. *Stroke* 36 1954–1959. 10.1161/01.STR.0000177517.01203.eb 16051896

[B214] Ueno H. Suemitsu S. Murakami S. Kitamura N. Wani K. Matsumoto Y. (2019). Layer-specific expression of extracellular matrix molecules in the mouse somatosensory and piriform cortices. *IBRO Rep.* 6 1–17. 10.1016/j.ibror.2018.11.006 30582064 PMC6293036

[B215] Ueno H. Suemitsu S. Murakami S. Kitamura N. Wani K. Okamoto M. (2017). Postnatal development of GABAergic interneurons and perineuronal nets in mouse temporal cortex subregions. *Int. J. Dev. Neurosci.* 63 27–37. 10.1016/j.ijdevneu.2017.08.003 28859888

[B216] Ueno H. Takao K. Suemitsu S. Murakami S. Kitamura N. Wani K. (2018). Age-dependent and region-specific alteration of parvalbumin neurons and perineuronal nets in the mouse cerebral cortex. *Neurochem. Int.* 112 59–70. 10.1016/j.neuint.2017.11.001 29126935

[B217] Valeri J. O’Donovan S. Wang W. Sinclair D. Bollavarapu R. Gisabella B. (2022). Altered expression of somatostatin signaling molecules and clock genes in the hippocampus of subjects with substance use disorder. *Front. Neurosci.* 16:903941. 10.3389/fnins.2022.903941 36161151 PMC9489843

[B218] Van den Oever M. Lubbers B. Goriounova N. Li K. Van der Schors R. Loos M. (2010). Extracellular matrix plasticity and GABAergic inhibition of prefrontal cortex pyramidal cells facilitates relapse to heroin seeking. *Neuropsychopharmacology* 35 2120–2133. 10.1038/npp.2010.90 20592718 PMC3055295

[B219] Van Dyken P. Lacoste B. (2018). Impact of metabolic syndrome on neuroinflammation and the blood-brain barrier. *Front. Neurosci.* 12:930. 10.3389/fnins.2018.00930 30618559 PMC6297847

[B220] Vazquez-Sanroman D. Leto K. Cerezo-Garcia M. Carbo-Gas M. Sanchis-Segura C. Carulli D. (2015). The cerebellum on cocaine: plasticity and metaplasticity. *Addict. Biol.* 20 941–955. 10.1111/adb.12223 25619460

[B221] Vazquez-Sanroman D. Monje R. Bardo M. (2017). Nicotine self-administration remodels perineuronal nets in ventral tegmental area and orbitofrontal cortex in adult male rats. *Addict. Biol.* 22 1743–1755. 10.1111/adb.12437 27549591 PMC5322253

[B222] Venturino A. Siegert S. (2021). Minimally invasive protocols and quantification for microglia-mediated perineuronal net disassembly in mouse brain. *STAR Protoc.* 2:101012. 10.1016/j.xpro.2021.101012 34950889 PMC8672101

[B223] Venturino A. Schulz R. De Jesus-Cortes H. Maes M. Nagy B. Reilly-Andujar F. (2021). Microglia enable mature perineuronal nets disassembly upon anesthetic ketamine exposure or 60-Hz light entrainment in the healthy brain. *Cell Rep.* 36:109313. 10.1016/j.celrep.2021.109313 34233180 PMC8284881

[B224] Volkow N. Wang G. Tomasi D. Baler R. (2013a). Obesity and addiction: neurobiological overlaps. *Obes. Rev.* 14 2–18. 10.1111/j.1467-789X.2012.01031.x 23016694 PMC4827343

[B225] Volkow N. Wang G. Tomasi D. Baler R. (2013b). The addictive dimensionality of obesity. *Biol. Psychiatry* 73 811–818. 10.1016/j.biopsych.2012.12.020 23374642 PMC4827347

[B226] Vujic T. Schvartz D. Furlani I. Meister I. Gonzalez-Ruiz V. Rudaz S. (2022). Oxidative stress and extracellular matrix remodeling are signature pathways of extracellular vesicles released upon morphine exposure on human brain microvascular endothelial cells. *Cells* 11:3926. 10.3390/cells11233926 36497184 PMC9741159

[B227] Wang H. Katagiri Y. McCann T. Unsworth E. Goldsmith P. Yu Z. (2008). Chondroitin-4-sulfation negatively regulates axonal guidance and growth. *J. Cell Sci.* 121(Pt. 18) 3083–3091. 10.1242/jcs.032649 18768934 PMC2562295

[B228] Warlow S. Robinson M. Berridge K. (2017). Optogenetic central amygdala stimulation intensifies and narrows motivation for cocaine. *J. Neurosci.* 37 8330–8348. 10.1523/JNEUROSCI.3141-16.2017 28751460 PMC5577851

[B229] Wei J. Lambert T. Y. Valada A. Patel N. Walker K. Lenders J. (2023). Single nucleus transcriptomics reveals pervasive glial activation in opioid overdose cases. *bioRxiv [Preprint]*. 10.1101/2023.03.07.531400 36945611 PMC10028861

[B230] Weiss F. (2005). Neurobiology of craving, conditioned reward and relapse. *Curr. Opin. Pharmacol.* 5 9–19. 10.1016/j.coph.2004.11.001 15661620

[B231] Wiggins A. Pacchioni A. Kalivas P. (2009). Integrin expression is altered after acute and chronic cocaine. *Neurosci. Lett.* 450 321–323. 10.1016/j.neulet.2008.12.006 19073234 PMC2646380

[B232] Willis A. Pratt J. Morris B. (2022). Enzymatic degradation of cortical perineuronal nets reverses GABAergic interneuron maturation. *Mol. Neurobiol.* 59 2874–2893. 10.1007/s12035-022-02772-z 35233718 PMC9016038

[B233] Wingert J. Sorg B. (2021). Impact of perineuronal nets on electrophysiology of parvalbumin interneurons, principal neurons, and brain oscillations: a review. *Front. Synaptic Neurosci.* 13:673210. 10.3389/fnsyn.2021.673210 34040511 PMC8141737

[B234] Wright J. Masino A. Reichert J. Turner G. Meighan S. Meighan P. (2003). Ethanol-induced impairment of spatial memory and brain matrix metalloproteinases. *Brain Res.* 963 252–261. 10.1016/s0006-8993(02)04036-2 12560131

[B235] Wu L. Ghitza U. Zhu H. Spratt S. Swartz M. Mannelli P. (2018). Substance use disorders and medical comorbidities among high-need, high-risk patients with diabetes. *Drug Alcohol Depend.* 186 86–93. 10.1016/j.drugalcdep.2018.01.008 29554592 PMC5959045

[B236] Xiong T. Wang X. Zha Y. Wang Y. (2022). Interleukin-33 regulates the functional state of microglia. *Front. Cell. Neurosci.* 16:1012968. 10.3389/fncel.2022.1012968 36439205 PMC9684324

[B237] Xue Y. Xue L. Liu J. He J. Deng J. Sun S. (2014). Depletion of perineuronal nets in the amygdala to enhance the erasure of drug memories. *J. Neurosci.* 34 6647–6658. 10.1523/JNEUROSCI.5390-13.2014 24806690 PMC6608143

[B238] Yin L. Feng R. Xie X. Yang X. Yang Z. Hu J. (2023). Matrix metalloproteinase-9 overexpression in the hippocampus reduces alcohol-induced conditioned-place preference by regulating synaptic plasticity in mice. *Behav. Brain Res.* 442:114330. 10.1016/j.bbr.2023.114330 36746309

[B239] Zarate C. Jr. Singh J. Carlson P. Brutsche N. Ameli R. Luckenbaugh D. (2006). A randomized trial of an N-methyl-D-aspartate antagonist in treatment-resistant major depression. *Arch. Gen. Psychiatry* 63 856–864. 10.1001/archpsyc.63.8.856 16894061

[B240] Zhang N. Yan Z. Liu H. Yu M. He Y. Liu H. (2021). Hypothalamic perineuronal nets are regulated by sex and dietary interventions. *Front Physiol.* 12:714104. 10.3389/fphys.2021.714104 34393830 PMC8355523

[B241] Zhang S. Zhornitsky S. Angarita G. Li C. (2020). Hypothalamic response to cocaine cues and cocaine addiction severity. *Addict. Biol.* 25:e12682. 10.1111/adb.12682 30295396 PMC6453736

[B242] Zironi I. Burattini C. Aicardi G. Janak P. (2006). Context is a trigger for relapse to alcohol. *Behav. Brain Res.* 167 150–155. 10.1016/j.bbr.2005.09.007 16256214

[B243] Zuo L. Gelernter J. Zhang C. Zhao H. Lu L. Kranzler H. (2012). Genome-wide association study of alcohol dependence implicates KIAA0040 on chromosome 1q. *Neuropsychopharmacology* 37 557–566. 10.1038/npp.2011.229 21956439 PMC3242317

